# Type I restriction–modification systems regulate gene expression through DNA topology

**DOI:** 10.1093/nar/gkag944

**Published:** 2026-09-30

**Authors:** Yasmeen Althari, Yongkui K Chen, Anna Menon, María José Ferrándiz, Renske van Raaphorst, Megan De Ste Croix, Andrew T Ulijasz, David G Glanville, Jan-Willem Veening, Stephen D Bentley, Nicholas J Croucher, Christopher D Bayliss, Adela G de la Campa, Jing-Ren Zhang, Claudia Trappetti, Richard D Haigh, Marco R Oggioni

**Affiliations:** Department of Genetics, Genomics and Cancer Sciences, University of Leicester, Leicester LE1 7RH, United Kingdom; College of Applied Medical Science, Jouf University, Aljouf 72388, Saudi Arabia; Department of Genetics, Genomics and Cancer Sciences, University of Leicester, Leicester LE1 7RH, United Kingdom; State Key Laboratory of Microbial Metabolism, Joint International Laboratory on Metabolic & Developmental Sciences, School of Life Sciences & Biotechnology, Shanghai Jiao Tong University, Shanghai 200240, China; Dipartimento di Farmacia e Biotecnologie, Università di Bologna, Bologna 40126, Italy; Unidad de Genética Bacteriana, Centro Nacional de Microbiología, Instituto de Salud Carlos III, Majadahonda, 28222 Madrid, Spain; University of Lausanne, Department of Fundamental Microbiology, Lausanne 1015, Switzerland; Department of Genetics, Genomics and Cancer Sciences, University of Leicester, Leicester LE1 7RH, United Kingdom; University of Alabama at Birmingham, Department of Medicine, Division of Pulmonary, Allergy and Critical Care Medicine, Birmingham, AL 35233, United States; University of Alabama at Birmingham, Department of Medicine, Division of Pulmonary, Allergy and Critical Care Medicine, Birmingham, AL 35233, United States; University of Lausanne, Department of Fundamental Microbiology, Lausanne 1015, Switzerland; Wellcome Sanger Institute, Parasites and Microbes, Hinxton CB10 1RQ, United Kingdom; Wellcome Sanger Institute, Parasites and Microbes, Hinxton CB10 1RQ, United Kingdom; MRC Centre for Global Infectious Disease Analysis, School of Public Health, Imperial College London, London W12 0BZ, United Kingdom; Department of Genetics, Genomics and Cancer Sciences, University of Leicester, Leicester LE1 7RH, United Kingdom; Unidad de Genética Bacteriana, Centro Nacional de Microbiología, Instituto de Salud Carlos III, Majadahonda, 28222 Madrid, Spain; Secretaría General, Consejo Superior de Investigaciones Científicas, 28040 Madrid, Spain; Tsinghua University, School of Basic Medical Sciences, Beijing 100084, China; Dipartimento di Farmacia e Biotecnologie, Università di Bologna, Bologna 40126, Italy; Saint Camillus International University of Health and Medical Sciences, 00131 Roma, Italy; Department of Genetics, Genomics and Cancer Sciences, University of Leicester, Leicester LE1 7RH, United Kingdom; Dipartimento di Farmacia e Biotecnologie, Università di Bologna, Bologna 40126, Italy; Platform RESTORE, IRCCS Azienda Ospedaliero-Universitaria di Bologna, Bologna, Italy

## Abstract

This study examines how DNA methylation by both phase-variable and canonical restriction–modification (RM) systems contributes to bacterial gene regulation, using the SpnD39III system of *Streptococcus pneumoniae* and the prototypical EcoKI system of *Escherichia coli*. RNA-seq analysis of pneumococcal phase-locked strains expressing individual SpnD39III specificities revealed widespread but promoter-context-dependent differential gene expression associated with methylation. Targeted luciferase reporter assays confirmed that methylation of specific intergenic sites can either activate or repress transcription, validating the transcriptomic observations. Genetic dissection demonstrated that these regulatory effects depend on methyltransferase activity mediated by HsdM and are independent of restriction endonuclease function. These methylation-dependent responses were conserved across pneumococcal strain backgrounds. Mechanistic analyses revealed that methylation alters DNA topology, with methylated plasmids exhibiting reduced negative supercoiling. Consistent with a topology-driven mechanism, modulation of the nucleoid-associated protein HlpA (HU) reshaped promoter responses in a manner dependent on methylation state and promoter topological responsiveness. Extension of these analyses to *E. coli* showed analogous methylation-dependent regulation by EcoKI, supported by reporter assays, quantitative polymerase chain reaction, RNA-seq, and methylome profiling. Together, these findings demonstrate that RM system-mediated DNA methylation functions as an epigenetic regulator across bacterial species by altering local DNA topology and promoter accessibility, thereby dynamically shaping bacterial gene regulatory networks.

## Graphical Abstract

**Graphical Abstract:**
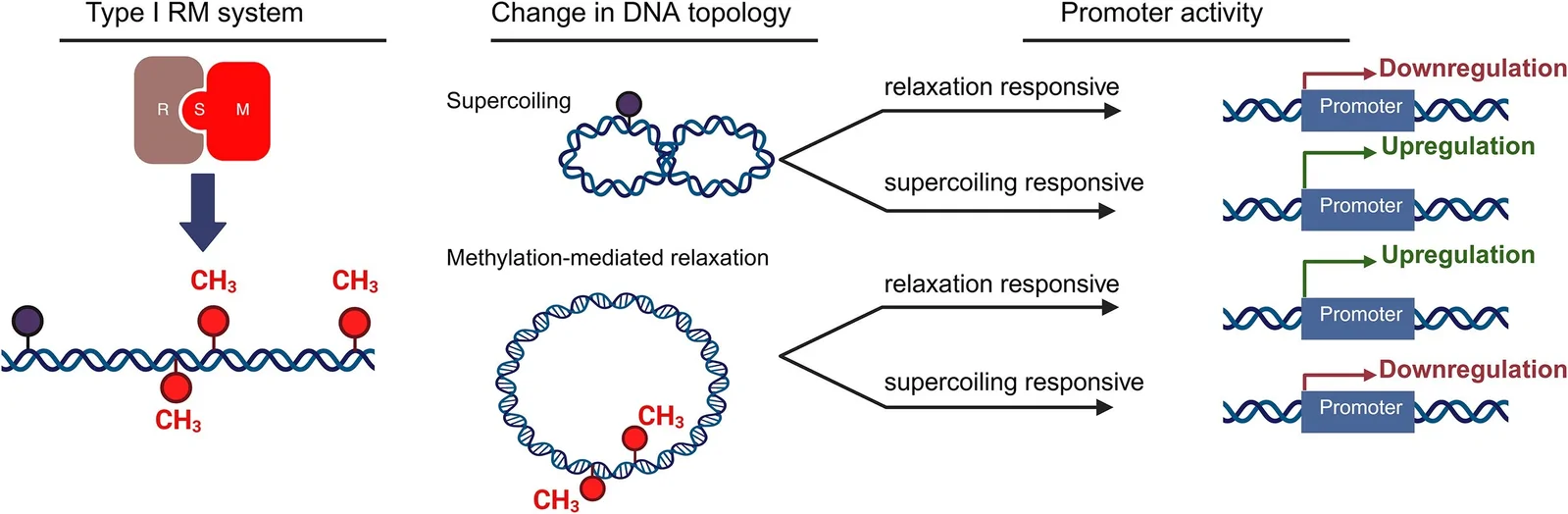

## Introduction

Epigenetics refers to the phenomenon of heritable but non-genetic regulation of gene expression. This is well established in eukaryotes, where it typically involves DNA methylation or, more commonly, histone modification [1]. In bacteria, epigenetics was long considered rare, limited to a few isolated cases of methylation-based control [2]. One of the best-studied bacterial DNA methyltransferases is the DNA adenine methyltransferase (Dam) of the *Gammaproteobacteria*, which methylates adenine in the GATC sequence. Beyond its roles in mismatch repair and replication initiation, Dam also regulates the expression of a limited set of genes [3]. Another example is the cell-cycle regulator CcrM of *Caulobacter crescentus*, which controls the transcription of multiple essential genes [4, 5].

In addition to these ‘orphan’ methyltransferases, most bacteria carry multiple methyltransferases encoded within their restriction–modification (RM) systems. RM systems couple an endonuclease and a methyltransferase, targeting identical recognition sequences to distinguish ‘self’ from foreign DNA. Being specific to phylogenetically closely related isolates, RM systems provide protection against horizontal gene transfer and phage infection, but at the cost of needing to methylate the host genome [6, 7]. Thus, RM systems act both as defensive barriers and as potential regulators of the host chromosome.

A striking feature of many Type I and III RM systems is phase variation (PV), in which stochastic changes alter either enzyme expression or the sequence specificity of the recognition subunit. PV increases bacterial fitness by reducing phage susceptibility and limiting DNA uptake [8–10]. A landmark discovery over 20 years ago showed that phase-variable RM systems can also alter the expression of multiple chromosomal genes, creating a ‘phasevarion’, a phase-variable regulon controlled epigenetically [11].

Despite the prevalence of phasevarions, only a handful of studies have identified the direct mechanistic basis for gene regulation by RM-mediated methylation. For example, autoregulation of the CfrBI RM system in *Citrobacter freundii* occurs through methylation of its own promoter [12]. More recently, direct promoter methylation was shown to regulate the *flaA* gene in *Helicobacter pylori* [13] and the *eda* gene in *Neisseria* [14]. This highlights a key paradox: although RM systems can methylate hundreds or thousands of sites in a genome, relatively few of these events have been linked to specific regulatory outcomes.

The mechanistic basis of methylation-dependent regulation can involve interference with DNA-binding proteins. For Dam, CcrM, and CfrBI, methylation alters the binding of transcription factors or sigma factors at defined promoters [12, 15]. In other cases, methylation inhibits binding of a repressor, thereby relieving transcriptional repression, as shown for CcrM effects on *ftsZ* and *mipZ* [5, 16] and for the DnmA methyltransferase in *Bacillus subtilis* [17]. Such examples suggest that DNA methylation can either block or facilitate protein–DNA interactions in a promoter-specific manner.

Here, we investigate the mechanistic regulation of gene expression by two Type I RM systems: the phase-variable SpnD39III system of *Streptococcus pneumoniae* [10, 18–22] and the non-phase-variable prototype EcoKI system of *Escherichia coli* [23, 24]. Type I systems comprise three genes: *hsdS*, encoding the DNA specificity subunit; *hsdM*, encoding the methyltransferase; and *hsdR*, encoding the restriction endonuclease. The HsdS and HsdM proteins form a site-specific methyltransferase, while HsdS/M/R together form the restriction enzyme complex. The cognate EcoKI enzyme recognizes the sequence (AACN_6_GTGC) [25]. In SpnD39III, site-specific recombination within the *hsdS* gene generates six allelic variants, each specifying a distinct recognition sequence [10].

The aim of this work was to test how SpnD39III and EcoKI methylation influence the expression of selected genes, thereby exploring whether Type I RM systems can provide a generalizable mechanism of epigenetic gene regulation in bacteria.

## Materials and methods

### Bacterial strains and growth conditions

All bacterial strains and plasmids used in this project are listed in Supplementary Tables S1 and S2. *Streptococcus pneumoniae* strains were grown statically at 37°C with 5% CO_2_ in Brain Heart Infusion (BHI; Oxoid, UK), Trypticase Soy Broth (Becton Dickinson, USA), or on BHI agar supplemented with 3% (v/v) horse blood (Thermo Scientific, UK). *Escherichia coli* strains were grown at 37°C with shaking in Luria–Bertani (LB) broth or on LB agar. Stocks were stored at −80°C in 10% (v/v) glycerol. Antibiotics were used as required: kanamycin (500 µg/ml), streptomycin (500 µg/ml), spectinomycin (200 µg/ml), ampicillin (100 µg/ml), and novobiocin (10 µg/ml).

### RNA sequencing and differential expression analysis

RNA sequencing was performed by Oxford Genomics using the Illumina NovaSeq 6000 platform. Reads were quality filtered and trimmed using Trimmomatic (v0.32). Processed reads were analysed using Rockhopper v2.0.3, which performs read alignment, transcript assembly, and expression quantification. Analyses were conducted using the reference genomes of *S. pneumoniae* D39 (GenBank NC_008533.2) or *E. coli* MG1655 (GenBank U00096). Differential expression was assessed by pairwise comparison of methylation-active and methylation-deficient strains. Genes were considered differentially expressed using a threshold of ≥2-fold change (|log_2_ fold change| ≥ 1) and adjusted *P* ≤ .01. Normalized expression values were derived from mapped reads. Data are deposited in GEO under accessions GSE293985 and GSE328962.

### Genome sequence annotation

Promoter architecture and transcriptional features were obtained from PneumoBrowse for *S. pneumoniae* D39[26], and EcoCyc for *E. coli* MG1655 [27].

### PCR and quantitative PCR

Polymerase chain reaction (PCR) was performed using Phusion High-Fidelity DNA polymerase (Thermo Scientific). Primers are listed in Supplementary Table S3. RNA was extracted using the Norgen Total RNA Kit or the Maxwell system (Promega) and treated with TURBO DNase. Complementary DNA synthesis and qPCR were performed from three independent cultures per condition using Fast SYBR Green Master Mix (Applied Biosystems) on a 7500 Real-Time PCR System. Relative expression was calculated using the comparative method [28], with expression normalized to the chromosomal reference gene *gyrB* in *S. pneumoniae* or *dxs* in *E. coli* [29]. Primers targeting the luciferase reporter and reference genes are listed in Supplementary Table S3.

### Plasmid copy number determination

Copy number of the EcoKI reporter plasmid pMRO893 was determined by SYBR Green qPCR using both absolute and relative quantification [29]. The plasmid-encoded *bla* gene was quantified relative to the chromosomal single-copy *dxs* gene. Both methods yielded consistent estimates, indicating a low copy number of ∼5–6 plasmids per chromosome.

### Construction of promoter-reporter variants

For both pneumococcal and *E. coli* assays, promoters were tested as wild-type (WT) sequences containing native Type I RM recognition sites, alongside sequence-modified variants in which sites were altered to match alternative specificities or abolished entirely. This strategy allowed discrimination between methylation-dependent effects and non-specific sequence changes. In *S. pneumoniae*, promoter–reporter strains were generated using Janus cassette counter-selection [30, 31] in phase-locked *spnD39III* backgrounds [10]. Synthetic DNA blocks (Thermo Fisher, UK) containing promoter variants flanked by ∼500 bp homology arms were used for allelic replacement (supplementary). In *E. coli*, promoter variants were cloned upstream of the *lux* operon using cloning sites in the pMRO893 low-copy reporter plasmid (supplementary).

In-frame deletions of *hsdM* were generated in MG1655 using the *sacB*-based suicide vector pRDH10 and allelic exchange, yielding unmarked mutants. The Δ*hsdM* genotype was confirmed by whole-genome sequencing.

### Transformation protocols

Natural transformation of *S. pneumoniae* was performed using CSP1-induced competence in BHI-CTM medium (Claverys, 2006). *Escherichia coli* transformations were performed by electroporation using a Bio-Rad Gene Pulser™ under standard conditions.

### Luminescence assays

Pneumococcal strains were grown overnight on blood agar, resuspended to OD_600_ = 0.1, diluted 1:10, and assayed in triplicate (technical and biological) in white 96-well plates. Luminescence was measured over 12 h following addition of catalase and luciferin using an Omega Bio-tek plate reader. ΔHU pMVHU strains were grown in chemically defined medium [32] supplemented with chloramphenicol and tetracycline; *hlpA* expression was induced with 0.8% maltose or repressed with 0.8% sucrose [33]. *Escherichia coli* luminescence assays were performed in LB medium without luciferin supplementation. For analysis, maximum luminescence during mid-log phase was extracted for each biological replicate and analysed as described under ‘Statistical analysis’ section.

### Whole-genome and methylome sequencing

Genomic DNA was extracted using the Genomic DNA Clean & Concentrator™ kit (Zymo Research). SMRT sequencing was performed at the Wellcome Trust Centre for Human Genetics (University of Oxford) using the PacBio Sequel II platform for pneumococcal phase-locked strains and for *E. coli* MG1655, MG1655 Δ*hsdM*, and DH5α. Processed methylation motif tables were used for downstream analyses and are provided as supplementary data. Genome assemblies have been deposited under BioProject accession number PRJNA1416954.

### Plasmid supercoiling density

To assess the effect of SpnD39III methylation on DNA topology, a recombinant pDP1 plasmid containing six additional SpnD39IIIE recognition sites and a kanamycin resistance cassette was constructed and introduced into *S. pneumoniae* R6, a D39 derivative lacking native pDP1. Plasmids were recovered from strains expressing locked SpnD39IIIE (methylated) or SpnD39IIIB (non-methylated) alleles. Supercoiling was analysed by two-dimensional agarose electrophoresis with chloroquine [34]. Superhelical density (σ) was calculated as ΔLk/Lk₀ (Lk₀ = plasmid size/10.5). Three biological replicates were analysed per strain.

### Statistical analysis

Statistical analyses were performed using GraphPad Prism v9. Luminescence data were log_10_-transformed prior to analysis. Two-group comparisons used unpaired Welch’s *t*-tests; multi-group comparisons used one-way analysis of variance (ANOVA) with Tukey’s post hoc test. Data are presented as mean ± SD. *P* < .05 was considered significant.

## Results

### Genome-wide methylome analysis confirms allele-specific methylation in SpnD39III phase-locked strains

The SpnD39III RM system of *S. pneumoniae* encodes six alternative HsdS subunits (HsdSA–F), each conferring a distinct DNA recognition specificity (Fig. 1A). To remove stochastic switching, we utilized ‘phase-locked’ strains; in which all but one *hsdS* allele were deleted [10]. These provide a controlled background for testing the effects of individual SpnD39III variant.

**Figure 1.**
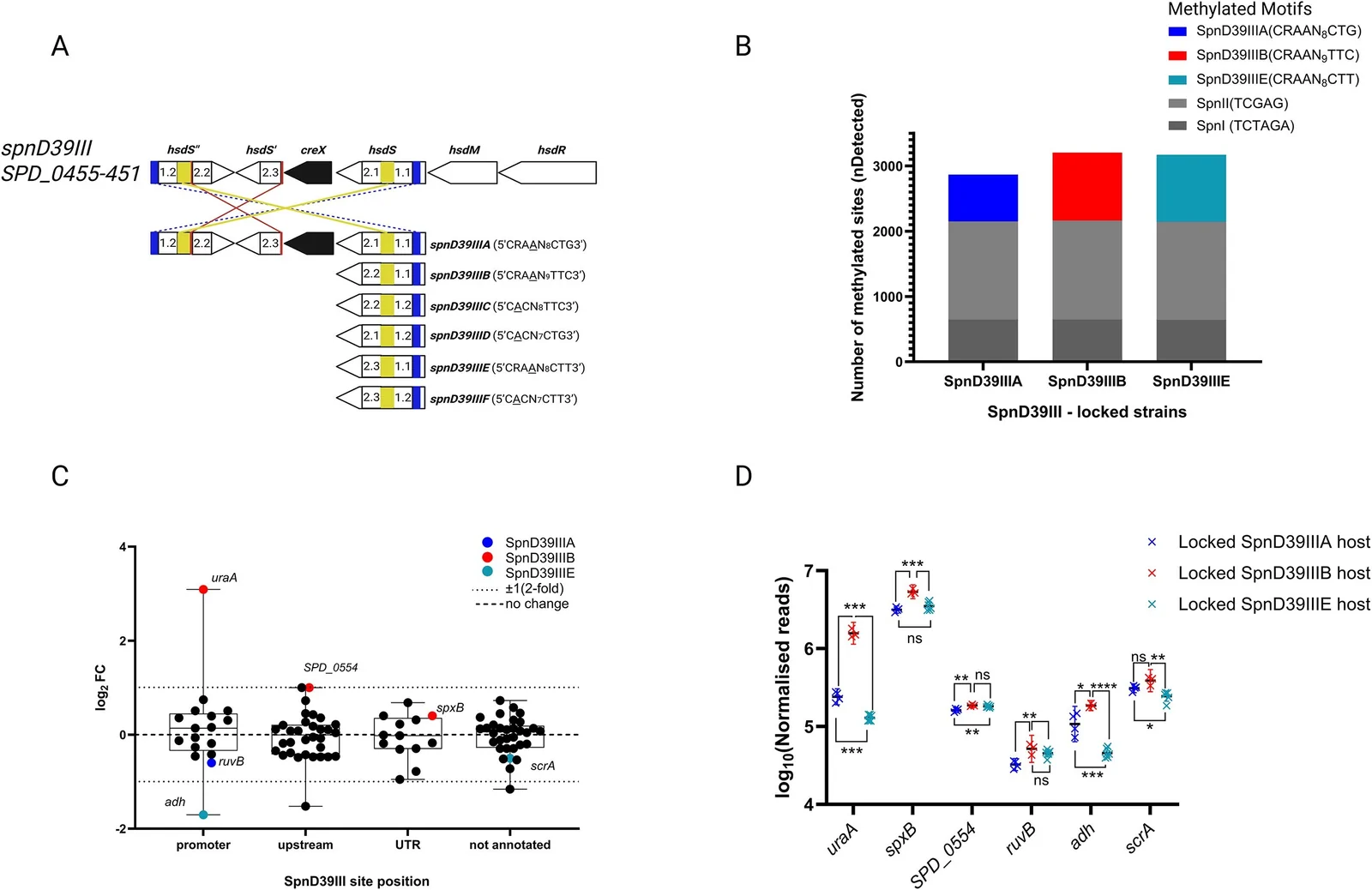
Structure of the SpnD39III Type I RM system and effects on gene expression. (**A**) Organization of the spnD39III locus in *S. pneumoniae* D39. The system encodes *hsdR* (SPD_0455), *hsdM* (SPD_0454), and hsdS (SPD_0453), together with a recombinase *creX* (SPD_0452) and truncated *hsdS* genes (*hsdS’, hsdSʺ*). Coloured boxes indicate inverted repeats that mediate recombination of target recognition domains. (**B**) RNA-seq analysis of 148 differentially expressed genes (Supplementary Table S4) containing SpnD39III recognition sites in their upstream intergenic regions. log_2_ fold change values show expression in the methylated host relative to the mean expression of two non-methylated hosts. Genes are grouped by the site location [promoter, upstream, untranslated region (UTR), unannotated]. Colours indicate DE genes with specificity of the site: SpnD39IIIA (blue), SpnD39IIIB (red), and SpnD39IIIE (green). The dashed line marks no change, and dotted lines denote ±1 (two-fold change). (**C**) Genome-wide methylome analysis of phase-locked SpnD39III strains. Bars show the total number of detected methylated sites (nDetected), partitioned into contributions from phase-variable SpnD39III specificities (blue, red, and green) and the core type II RMS motifs SpnII (TCGAG, grey) and SpnI (TCTAGA, dark grey). All sites were fully methylated (fraction = 1.0) for SpnD39III and SpnI motifs, while SpnII showed near-complete methylation (fraction = 0.997). (**D**) Expression of representative genes containing SpnD39III sites. RNA-seq read counts (log_10_ normalized values) are shown for phase-locked strains expressing SpnD39IIIA (blue, *n* = 4), SpnD39IIIB (red, *n* = 3), or SpnD39IIIE (green, *n* = 6). Points represent biological replicates; bars indicate mean ± SD. Statistical analysis was performed by one-way ANOVA followed by Tukey’s multiple comparisons test. Significance is indicated as: *P* < .05 (*), *P* < .01 (**), *P* < .001 (***), *P* < .0001 (****); ns, not significant. For image description, please refer to the figure legend and surrounding text.

To establish the genome-wide methylation landscape, we performed PacBio SMRT sequencing on strains phase-locked for the *spnD39IIIA, spnD39IIIB*, and *spnD39IIIE* alleles. In each background, methylation was detected exclusively at the cognate recognition motif, with no modification at non-cognate sites. For SpnD39IIIA (CRAAN_8_CTG), SpnD39IIIB (CRAAN_9_TTC), and SpnD39IIIE (CRAAN_8_CTT), >99% of predicted motifs were detected and fully methylated (fraction = 1.0), demonstrating complete penetrance of the locked alleles. In addition, the conserved Type II systems SpnI (TCTAGA) and SpnII (TCGAG) were consistently methylated in all strains, with fractions of 1.0 and 0.997, respectively (Supplementary Tables S8–S11) [10]. These data establish that strains locked for *spnD39III* alleles generate precise, allele-specific methylation patterns across the genome (Fig. 1B).

### Differential gene expression in phase-locked strains

To determine whether SpnD39III methylation influences transcription, we performed RNA-seq on strains phase-locked for *spnD39IIIA, spnD39IIIB*, or *spnD39IIIE* alleles. In total, 455 genes showed significant differential expression (Padj < 0.01). Of these, 148 carried at least one SpnD39III recognition site in the upstream intergenic region (Supplementary Table S4). Among these, 87 genes (19.1% of all differentially expressed genes) were linked specifically to the recognition sites of the three tested variants (SpnD39IIIA, Spn D39IIIB, or SpnD39IIIE). This highlights the potential of SpnD39III methylation to exert broad, transcriptome-wide effects.

Most of these changes in gene expression were modest, typically within a two-fold difference relative to the mean expression of the two non-methylated host strains (i.e. strains in which the recognition site was present but not methylated due to expression of a non-matching SpnD39III variant). Such modest shifts are consistent with methylation fine-tuning transcription rather than acting as an on/off switch. A notable exception was the *uraA* gene, which was strongly upregulated (eight-fold) in the SpnD39IIIB background (Fig. 1C), suggesting that in some cases SpnD39III methylation can exert a substantial regulatory effect.

### Methylation status determines promoter activity

To directly test whether methylation of SpnD39III sites drives transcriptional differences, we constructed luminescent reporter strains using the click beetle luciferase (*CBRluc*) gene. *CBRluc* reporter constructs were introduced into phase-locked pneumococcal strains expressing SpnD39IIIA, SpnD39IIIB, SpnD39IIIE, SpnD39IIIF, or an *spnD39III* knockout strain. Each construct contained ∼500 bp of flanking homology (upstream of SPD-0561 and downstream of *CBRluc*) to facilitate chromosomal integration (Supplementary Fig. S1) [35].

Synthetic promoter variants were generated for six genes identified by RNA-seq as reproducibly differentially expressed and containing intergenic SpnD39III sites (Fig. 1C and D). Three genes were upregulated in methylated hosts: the uracil–xanthine permease *uraA* (SPD_1141), the ABC transporter SPD_0554, which participates in pyrimidine metabolism [36], and the pyruvate oxidase *spxB* (SPD_0636) previously linked to opaque–transparent PV [37]. Three genes were downregulated: the Holliday junction helicase *ruvB* (SPD_0241), the zinc-dependent alcohol dehydrogenase *adh* (SPD_1865), and the sucrose-specific PTS component *scrA* (SPD_1532), which acts as a repressor of the *scr* operon [38].The locations of the SpnD39III sites were consistent with potential effects on transcription in *uraA, spxB, ruvB, SPD_0554*, and *adh*, where sites were positioned adjacent to the –35/–10 promoter elements, whereas in *scrA* the site mapped to the 5′UTR (supplementary).

Reporter assays validated the transcriptional trends identified by RNA-seq. Peak luminescence from the upregulated promoters was consistently higher when their WT sites were methylated by the cognate SpnD39III variant (Fig. 2A–C). In non-methylating backgrounds (different *spnD39III* allele or *spnD39III* knockout), activity decreased by up to 10-fold in the case of *uraA*. For the downregulated set, the *adh* and *scrA* promoters showed significantly lower expression in methylating host (SpnD39IIIE) compared to SpnD39IIIF hosts, while the *ruvB* promoter was more strongly expressed in the SpnD39IIIB background where its SpnD39IIIA site was not methylated (Fig. 2D–F).

**Figure 2.**
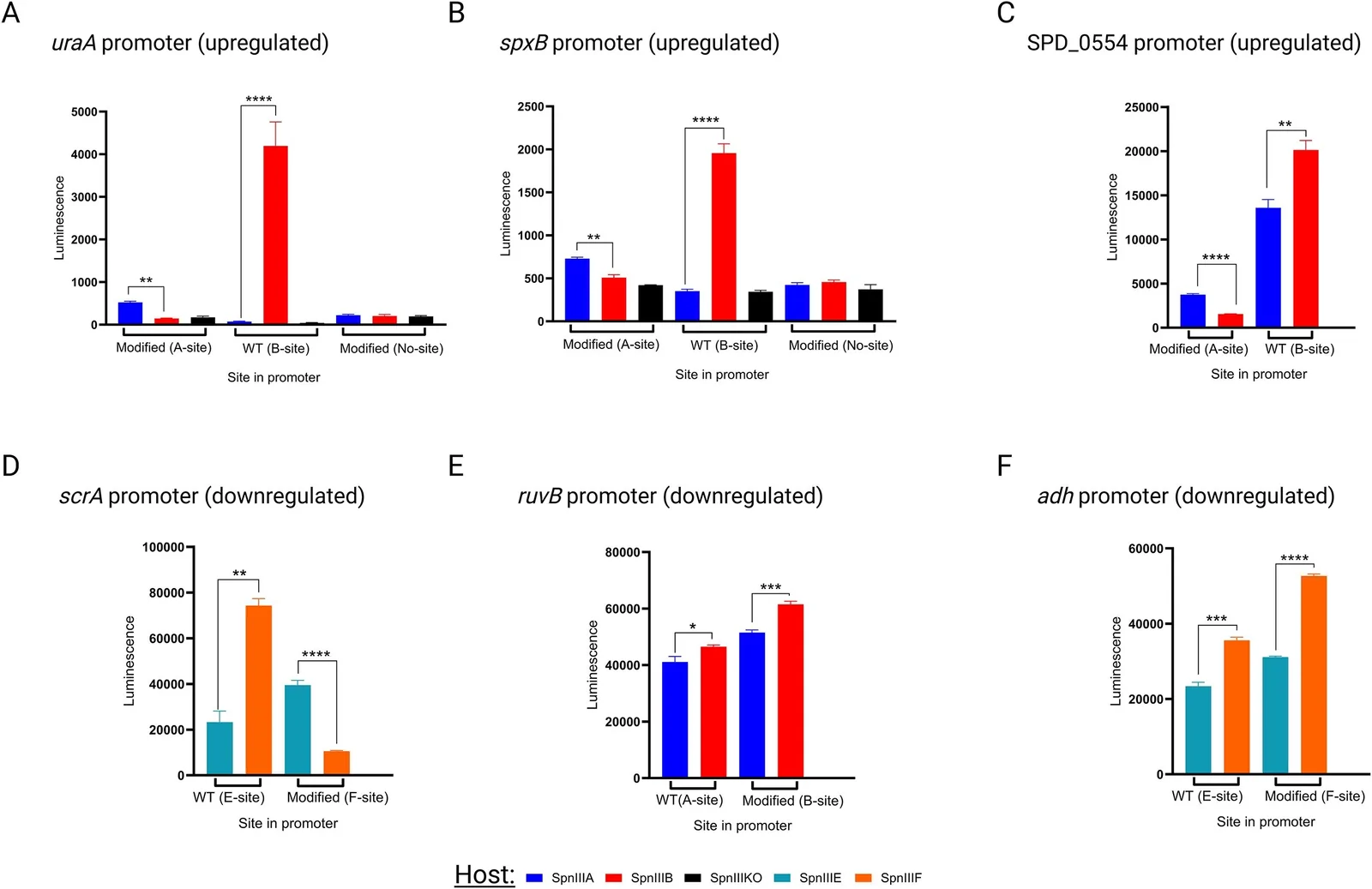
Differential reporter gene expression from pneumococcal promoters carrying intergenic SpnD39III recognition sites. (A–F) Maximum luminescence values from luciferase reporter assays for six promoters identified by RNA-seq as SpnD39III-regulated. Promoters upregulated in a methylating host: *uraA* (**A**), *spxB* (**B**), SPD_0554 (**C**). Promoters downregulated in a methylating host: *scrA* (**D**), *ruvB* (**E**), *adh* (**F**). For each promoter, WT constructs contained the native recognition site (e.g. *uraA* with a B-site), whereas modified constructs carried motif substitutions matching alternative SpnD39III specificities (e.g. A-site for *uraA*) or a complete deletion (No-site). Reporter constructs were tested in phase-locked hosts expressing distinct SpnD39III specificities (blue = SpnD39IIIA, red = SpnD39IIIB, green = SpnD39IIIE, orange = SpnD39IIIF), or in an SpnD39III knockout strain (black). Data are shown as mean ± SD of biological triplicates (*n* = 3 per construct). Raw luminescence values are plotted, with statistical analyses performed on log_10_-transformed values using unpaired two-tailed Welch’s *t*-tests to compare each construct in a methylating versus non-methylating host background. Significance is indicated as: *P* < .05 (*), *P* < .01 (**), *P* < .001 (***), *P* < .0001 (****), ns = not significant.

Point-mutated constructs with altered SpnD39III recognition sequence generally behaved as expected, showing higher expression in methylated backgrounds for upregulated promoters and lower expression in methylated backgrounds for downregulated promoters (Fig. 2 A–D). By contrast, constructs in which the SpnD39III site was abolished showed no differential expression in any host background, confirming that the regulatory effects depend specifically on the presence of a cognate site rather than non-specific sequence changes (Fig. 2 A and B). An exception was observed for *ruvB* and *adh*, where site-altering substitutions failed to reproduce the regulatory effect (Fig. 2 E and F). This suggests that the introduced mutations disrupted additional sequence features required for methylation sensitivity. Thus, the regulatory outcome depends not only on the presence of a methylated site but also on its precise sequence context.

In order to confirm that the alterations in luminescence observed in the reporter strains corresponded to changes in messenger RNA (mRNA) expression, additional experiments with larger-volume cultures were prepared. Luminescence from these cultures mirrored the results shown in Fig. 2 (data not shown). Importantly, RNA was extracted from the same cultures and *CBRluc* transcript levels were quantified by qPCR. qPCR analysis confirmed that *uraA* expression followed the same methylation-dependent trends observed in the luminescence assays, with significantly higher expression in matching methylating hosts than in non-methylating backgrounds (Fig. 3A).

**Figure 3.**
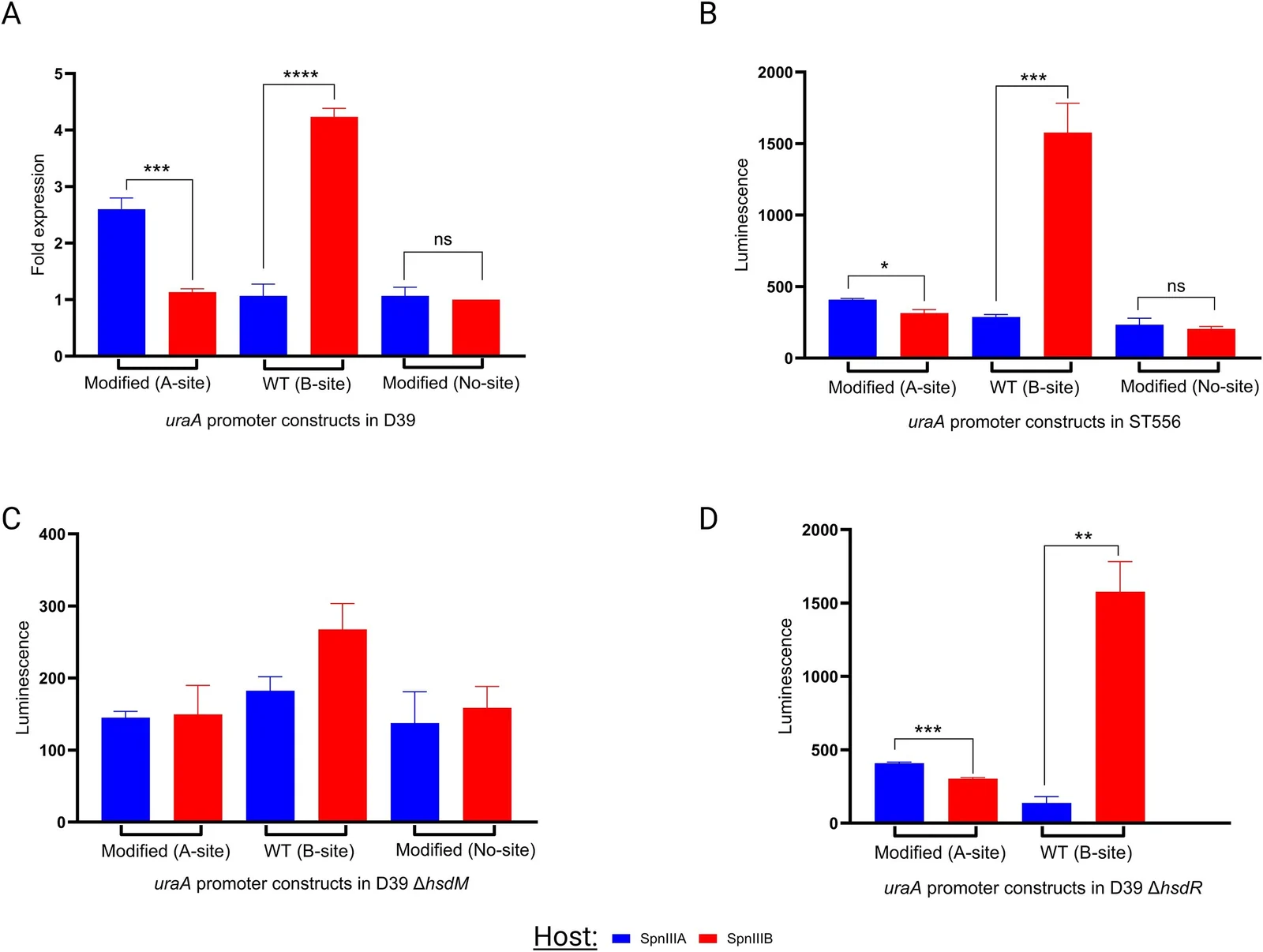
Methylation-dependent promoter regulation confirmed by qPCR, conserved across strains, and dependent on HsdM. (**A**) qPCR analysis of *uraA* promoter constructs in *S. pneumoniae* D39 reporter strains. Constructs contained a modified A-site, the WT B-site, or a site-deleted control. Expression of the *CBRluc* gene was normalized to *gyrB* and expressed relative to the no-site construct in the SpnD39IIIB strain (set to 1). Data are shown as mean ± SD of biological replicates. Statistical comparisons were performed using unpaired two-tailed *t*-tests. (**B**) Luminescence from the same *uraA* promoter constructs in ST556 strains phase-locked for SpnST556IIIA or SpnST556IIIB. (**C**) Luminescence from *uraA* promoter constructs in an *hsdM* frame-shift mutant background. (**D**) Luminescence from *uraA* promoter constructs in an *hsdR* frame-shift mutant background.

For panels (B–D), data represent raw luminescence values plotted as mean ± SD of biological replicates (*n* = 3 per construct/strain; see ‘Materials and methods’ section). Statistical analyses were performed on log_10_-transformed values using unpaired two-tailed Welch’s *t*-tests to compare methylating versus non-methylating conditions for each construct. Significance is indicated as: *P* < .05 (*), *P* < .01 (**), *P* < .001 (***), *P* < .0001 (****), ns = not significant. *Y*-axis scales differ between panels to reflect differences in transcript abundance or luminescence intensity.

### SpnD39III is part of the core genome, and its regulatory effects are conserved across strains

SpnD39III has previously been identified as a core genome locus in *S. pneumoniae* [39]. To test whether its regulatory effects are strain-specific, comparable reporter constructs were generated in the serotype 19F strain ST559, for which methylome data had confirmed that the SpnST559III system shares the target site specificities with the orthologue SpnD39III system (Li *et al*., 2019). As in D39, the *uraA* promoter constructs carrying a WT SpnST559IIIB site produced higher luminescence in SpnST559IIIB hosts, whereas constructs carrying an SpnST559IIIA site showed higher luminescence in SpnST559IIIA hosts (Fig. 3B). Promoters lacking an SpnST559III site displayed no differential expression. These results confirm that SpnIII-mediated regulation is conserved across pneumococcal strains and not restricted to a single genetic background.

### The HsdM subunit is required for SpnD39III-dependent promoter regulation

The complete SpnD39III complex is composed of one HsdS, two HsdM, and two HsdR subunits. To dissect which components are required for epigenetic regulation, promoter-*luc* constructs were introduced into *S. pneumoniae* D39 reporter strains carrying frame-shift mutations in either *hsdM* or *hsdR*. Frame-shift mutants were selected, rather than deletions, to minimize potential effects on transcript stability of the *hsdRMS* operon and consequent disruption of *hsdS* expression. In the *hsdM* mutant, where the HsdM subunit is disrupted and target methylation cannot occur, luminescence levels were abolished and no longer differed between WT and modified promoter constructs (Fig. 3C). This indicates that methylation is essential for promoter regulation and that loss of HsdM eliminates the regulatory effect, likely both by preventing methyltransferase activity and by destabilizing the RM complex. In contrast, the *hsdR* mutant retained differential reporter expression between promoter variants (Fig. 3D), demonstrating that the endonuclease activity of HsdR is not required for regulation.

### SpnD39III methylation alters DNA topology, and HU modulates promoter responsiveness

Genome-wide methylome analysis confirmed that all SpnD39III recognition sites in phase-locked pneumococcal strains were fully methylated. This provided a reliable foundation to test whether methylation affects DNA conformation. To directly examine the topological consequences of methylation, we used the pneumococcal plasmid pDP1 as a topological reporter [40]. A recombinant pDP1 derivative was engineered to contain nine SpnD39III sites (18 methylated adenines) in addition of nine SpnD39II sites (nine methylated adenines) (Fig. 4A). This recombinant plasmid was introduced into SpnD39III phase-locked *S. pneumoniae* R6 strain, a plasmid-free derivative of D39. SMRT sequencing of R6 locked for the *spnD39IIIE* allele, with or without the recombinant plasmid, confirmed complete methylation of all sites (Fig. 4B and Supplementary Tables S10 and S11).

**Figure 4.**
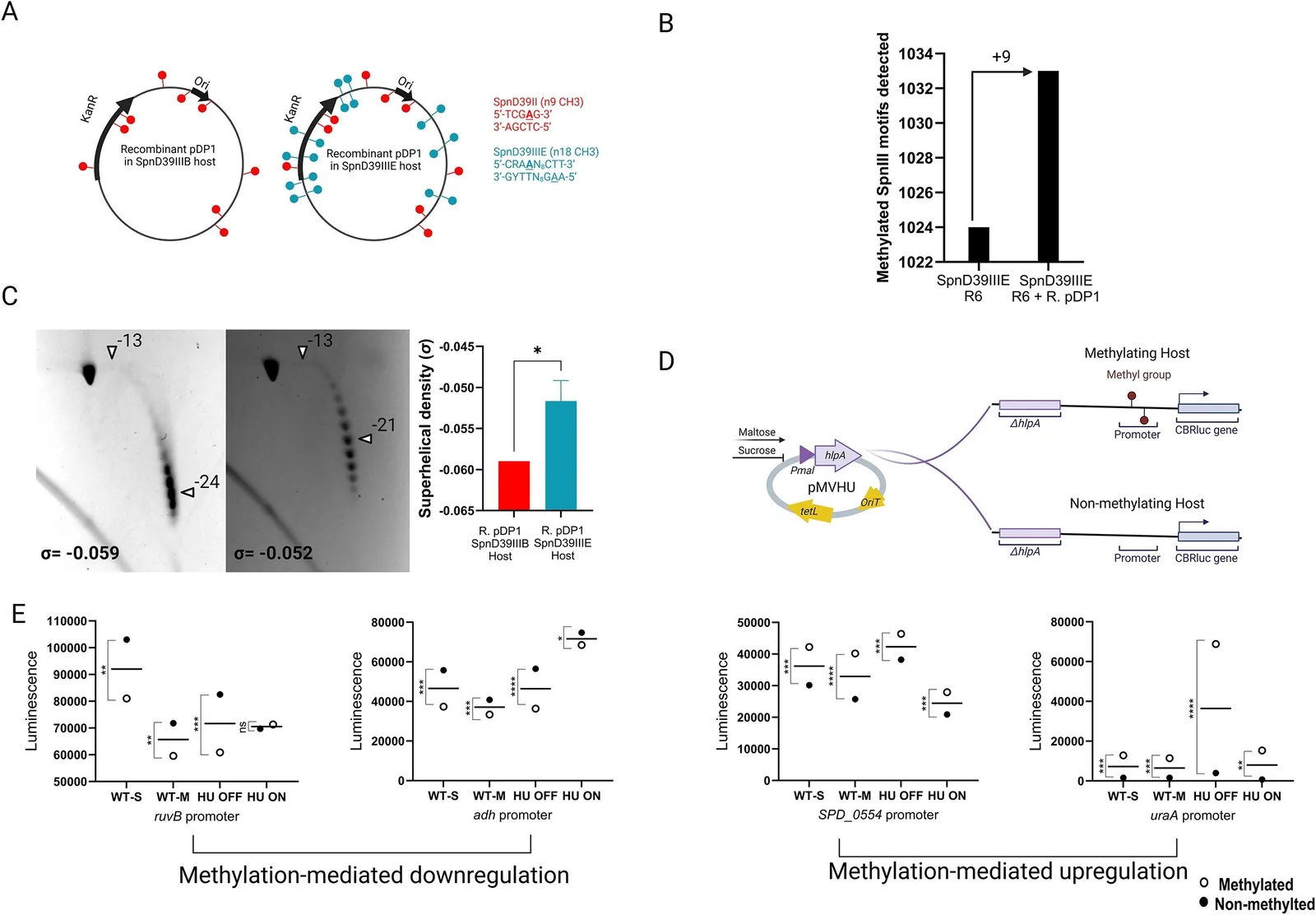
SpnD39III-dependent methylation modulates DNA topology and HU-responsive promoter activity. (**A**) Map of recombinant pDP1 (4241 bp) with nine SpnD39IIIE (green) and nine SpnD39II (red) sites. SpnD39IIIE = fully methylated, SpnD39II = hemi-methylated. The schematic illustrates the expected methylation pattern in *S. pneumoniae* R6 backgrounds expressing either the SpnD39IIIE or SpnD39IIIB specificities. (**B**) PacBio SMRT methylome of SpnD39IIIE phase-locked R6 strains ± recombinant pDP1, confirming complete SpnD39IIIE methylation. (**C**) Two-dimensional chloroquine gels of plasmids from R6 expressing SpnD39IIIB or SpnD39IIIE. Methylated plasmid more relaxed (σ ≈ −0.052 ± 0.003) versus non-methylated (σ ≈ −0.059 ± 0.000). Mean ± SD, *n* = 3; *P* < .05. (**D**) Schematic of HU modulation experiment. *hlpA* under maltose-inducible promoter on pMVHU in Δ*hlpA* strains. Growth in sucrose = HU OFF; maltose = HU ON. *CBRluc* reporters integrated to test methylation × HU effects. (**E**) Luminescence of methylation-activated (SPD_0554, *uraA*) and methylation-repressed (*ruvB, adh*) promoters under four HU conditions. White = methylating host, black = non-methylating. Mean ± SD, log_10_-transformed, Welch’s *t*-test, *P* < .05.

To evaluate the effect of SpnD39III methylation on plasmid supercoiling density, the recombinant plasmid was propagated in R6 strains expressing either SpnD39IIIE, generating a fully methylated plasmid, or SpnD39IIIB, resulting in plasmids methylated only at SpnD39II sites. Two-dimensional agarose electrophoresis revealed a consistent and reproducible decrease in superhelical density (∼12%) in methylated plasmids compared with non-methylated counterparts (Fig. 4C). Thus, SpnD39III-mediated methylation produces measurably more relaxed DNA, consistent with established links between local methylation, duplex stability, and torsional stress. Importantly, the same methylation-dependent shift in plasmid topology was observed when the native pDP1 plasmid, containing only its three endogenous SpnD39IIIE sites (Supplementary Fig. S3). These results indicate that SpnD39III methylation can alter the physical state of DNA, providing a plausible structural mechanism underlying promoter regulation.

Because nucleoid-associated proteins, particularly HU (HlpA), are major determinants of chromosomal architecture and supercoiling homeostasis in *S. pneumoniae* [33], we next asked whether HU contributes to methylation-dependent promoter regulation. We therefore used an inducible *hlpA* expression system (pMVHU), in which HU levels are reduced in sucrose (HU OFF) and increased in maltose (HU ON), to modulate DNA supercoiling *in vivo*. This system was used given the essentiality of *hlpA* in pneumococcus and the unfeasibility of its complete removal [33, 41, 42]. Four model promoters, two upregulated by SpnD39III methylation (*uraA*, SPD_0554) and two downregulated (*ruvB, adh*), were examined. In WT HU backgrounds, methylation produced the expected promoter-specific effects. However, altering HU levels modulated the magnitude and, in some cases, the direction of promoter activity in a manner consistent with each promoter’s supercoiling sensitivity.

For methylation-activated promoters (*uraA* and SPD_0554), HU depletion, which relaxes chromosomal DNA, markedly increased expression, whereas HU induction (increasing negative supercoiling) suppressed promoter activity (Fig. 4E). In contrast, methylation-repressed promoters (*ruvB* and *adh*) behaved as supercoiling-activated promoters: HU induction increased transcription and partially reversed the methylation effect, while HU repression reduced expression and accentuated the methylation-dependent repression (Fig. 4E). Importantly, in every condition the relative ordering of methylated versus non-methylated constructs was preserved; what changed was the amplitude of promoter activity as a function of DNA topology. Promoters known to be intrinsically sugar-responsive (*spxB, scrA*) were excluded from this analysis because maltose/sucrose-dependent regulation obscured the effects of HU or methylation.

Together, these findings support a unified model in which SpnD39III-dependent methylation modulates transcription by altering local DNA topology, producing promoter-specific regulatory outcomes based on intrinsic supercoiling sensitivity. HU does not distinguish methylated DNA via direct binding but instead amplifies or attenuates the transcriptional effects of methylation by globally tuning DNA supercoiling. Promoters upregulated by methylation behave as relaxation-activated loci, whereas those downregulated by methylation respond as supercoiling-activated loci. Together, these findings support a model in which SpnD39III-dependent methylation modulates DNA supercoiling and thereby alters promoter topology, influencing transcription in an HU-dependent manner.

### EcoKI methylation modulates promoter activity in *E. coli*, confirming cross-species conservation of Type I RMS-mediated epigenetic regulation

To determine whether methylation-dependent transcriptional effects observed for SpnD39III and SpnST559III in *S. pneumoniae* represent a broader property of Type I RMSs, we examined the prototypic EcoKI system of *E. coli*. The MG1655 genome contains 595 EcoKI recognition motifs (AACN_6_GTGC), including 20 intergenic sites, 11 of which map within predicted promoter regions (two overlapping − 35 elements and nine located in 5′UTRs). Four promoters containing such sites (*argR, yccA, rnc*, and *prpR*) were selected for further analysis. These promoters were selected based on the presence of EcoKI sites within predicted promoter regions, enabling direct assessment of methylation-dependent effects on transcription. The selected promoter regions were cloned into pMRO893, a low-copy luminescence reporter plasmid, either with the WT sequence or with the EcoKI site removed. The copy number of this reporter plasmid was experimentally verified by qPCR (≈5–6 copies per cell using *dxs* as the single-copy chromosomal reference).

Reporter constructs were tested in strains differing in EcoKI activity: MG1655 WT; an in-frame Δ*hsdM* deletion mutant of MG1655; and DH5α, which lacks HsdR retaining an intact HsdM (Fig. 5A). Initial comparison of MG1655 Δ*hsdR* with DH5α revealed no significant differences in promoter output; therefore, DH5α was used as the methylation-competent background for subsequent reporter assays because of its cloning efficiency. PacBio SMRT sequencing of MG1655 WT, MG1655 Δ*hsdM*, and DH5α confirmed complete methylation in the EcoKI-active strains and absence of methylation in the *ΔhsdM* mutant (Fig. 5B and Supplementary Tables S5–S7).

**Figure 5.**
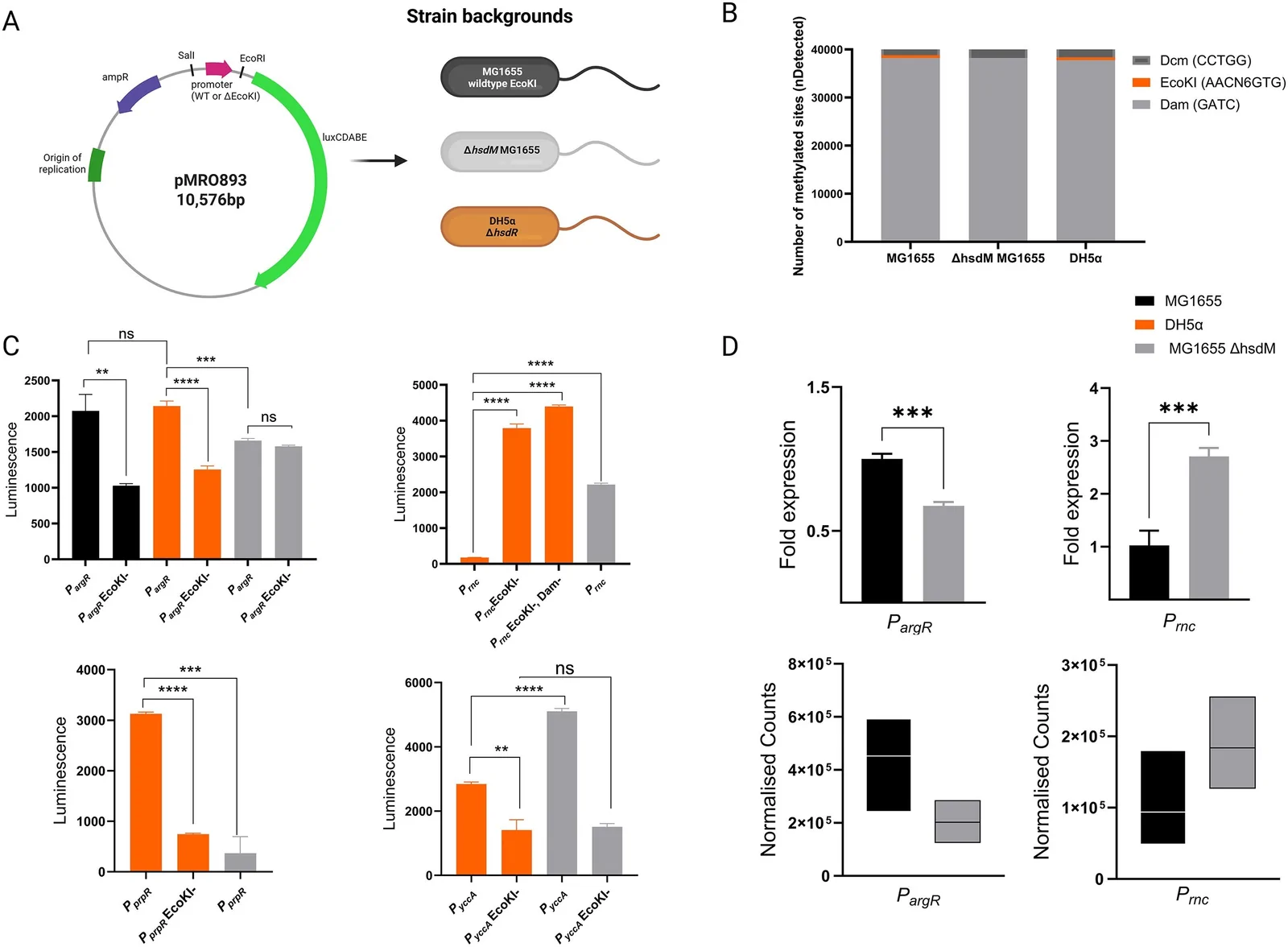
EcoKI methylation modulates promoter activity in *E. coli*. (**A**) Schematic of the pMRO893 low-copy reporter plasmid used to assess methylation-dependent promoter activity. The plasmid was introduced into three *E. coli* backgrounds with different EcoKI activities. (**B**) Genome-wide methylation profiles of EcoKI-proficient and -deficient hosts. Bars represent the total number of methylated motifs detected per genome. (**C**) Methylation-dependent effects of EcoKI on promoter activity using *lux* reporters. Maximum luminescence from WT and EcoKI-site–deleted variants of *argR, rnc, yccA*, and *prpR* promoters cloned into pMRO893. Reporter plasmids were assayed in EcoKI-active (DH5α, orange), EcoKI-inactive (MG1655 Δ*hsdM*, grey), and EcoKI-active control (MG1655, black). The *rnc* promoter was additionally tested with its internal Dam site removed. Luminescence was recorded from biological triplicates in mid-log phase; statistical analyses were performed on log_10_-transformed values using unpaired two-tailed Welch’s *t*-tests (methylating versus non-methylating). Significance is indicated as: *P* < .05 (*), *P* < .01(**), *P* < .001 (***), *P* < .0001 (****), ns = not significant. (**D**) qPCR and RNA-seq validation of methylation-dependent changes in transcription. (Top) qPCR quantification of *luxA* expression in MG1655 (WT EcoKI) and MG1655 Δ*hsdM* carrying *argR*–*lux* or *rnc*–*lux* constructs. Expression was normalized to the chromosomal *dxs* reference gene and analysed using the ΔΔCT method (unpaired *t*-test, *P* < .05). (Bottom) RNA-seq–derived expression levels of *argR* and *rnc* from MG1655 (WT EcoKI; black) and MG1655 Δ*hsdM* (EcoKI⁻; grey).

Luminescence assays demonstrated clear promoter-specific effects of EcoKI methylation. For *argR, yccA*, and *prpR*, WT promoters displayed substantially higher luminescence in WT MG1655 and/or DH5α than their corresponding no-site variants and were also expressed more strongly in DH5α than in the Δ*hsdM* mutant (Fig. 5C). For *prpR*, luminescence assays were performed in the presence of propionate to induce promoter activity, consistent with its known regulation by propionate availability [43]. In contrast, the *rnc* promoter showed the opposite behaviour: removal of the EcoKI site increased expression, and *rnc* activity was higher in the Δ*hsdM* mutant than in the methylation-competent DH5α (Fig. 5C). Thus, as observed for SpnD39III in pneumococcus, EcoKI methylation can either activate or repress transcription depending on the promoter context.

To validate the reporter output, *luxA* mRNA levels were quantified by qPCR for the *argR* and *rnc* constructs. In both cases, transcript abundance mirrored luminescence trends: *argR* expression was higher when the promoter was methylated, whereas *rnc* was more highly expressed in the methylation-deficient Δ*hsdM* mutant (Fig. 5D). These data confirmed that reporter luminescence reliably reflects transcriptional changes driven by EcoKI methylation.

To assess the broader transcriptional impact of EcoKI, RNA-seq was performed on MG1655 WT and the Δ*hsdM* mutant in biological triplicate. Differential expression analysis using a ≥2-fold change threshold (|log_2_FC| ≥ 1) and adjusted *P*-value ≤ .01 identified 99 significantly upregulated and 89 significantly downregulated genes in the *ΔhsdM* background. Functional enrichment analysis using the PANTHER classification system [44] indicated overrepresentation of genes involved in metabolic and catalytic pathways. Importantly, RNA-seq trends for the EcoKI-site-containing genes *argR* and *rnc* matched both our reporter assays and our qPCR data (Fig. 5D). Consistent with the requirement for propionate induction in the reporter assays, chromosomal *prpR* expression was negligible under the conditions used for RNA-seq and was therefore not considered suitable for direct comparison with the reporter assay data. Furthermore, re-examination of publicly deposited RNA-seq data from Mehershahi and Chen [45], although not analysed for these loci in their study, showed expression differences in the same direction, providing independent support for the EcoKI methylation effects observed here (Supplementary Fig. S2).

Collectively, these results demonstrate that EcoKI methylation produces promoter-specific transcriptional effects in *E. coli*, including both activation and repression, and that these effects depend on the presence of the MTase rather than the restriction subunit. The conserved behaviour of EcoKI and SpnD39III across species supports a general model in which Type I MTases modulate transcription through methylation-dependent structural effects on promoter architecture.

## Discussion

Phase-variable Type I RM systems have been recognized as an epigenetic regulator of bacterial gene expression, yet the underlying mechanism remains incompletely understood [46–49]. Here, we show that the pneumococcal SpnD39III system exerts promoter-specific regulatory effects that are mediated not by direct recruitment of regulatory proteins, but through changes in DNA topology.

By combining genome-wide methylome profiling, transcriptional analysis, plasmid supercoiling assays, and modulation of nucleoid-associated protein levels, we provide convergent evidence that SpnD39III methylation influences transcription by altering the topological state of DNA, with promoter responses shaped by intrinsic supercoiling sensitivity.

Genome-wide methylome analysis confirmed that phase-locked SpnD39III strains produce complete, allele-specific methylation of their cognate target sites across the chromosome. This full penetrance is critical, as it establishes that the transcriptional differences observed between phase-locked strains reflect stable epigenetic states rather than partial or heterogeneous methylation. Observed differential gene expression mediated by Type I RM systems is consistent with previous reports in pneumococcus, *H. influenzae, N. gonorrhoeae*, and *C. jejuni* [10, 11, 50, 51]. RNA-seq analysis revealed that while SpnD39III methylation affected a large number of genes, most changes were modest in magnitude, consistent with methylation acting as a fine-tuning mechanism rather than a binary on/off switch.

To link methylation to a physical mechanism, we exploited the small pneumococcal plasmid pDP1 as a topological reporter. Introduction of additional SpnD39IIIE sites into pDP1 resulted in a reproducible relaxation of plasmid supercoiling when methylated, compared with the same plasmid propagated in non-methylating hosts. Importantly, methylome sequencing confirmed that the increase in detected SpnD39IIIE motifs exactly matched the number of sites introduced, demonstrating complete methylation of the engineered plasmid and excluding indirect effects. These findings align with prior work showing that DNA methylation can alter helix geometry, stiffness, and torsional stress [52], and support a model in which SpnD39III methylation induces local changes in DNA topology that propagate to influence transcription. Together, our data indicate that methylation state is a critical determinant of the physical DNA environment, thereby shaping promoter accessibility and transcriptional output. Given that DNA topology in *S. pneumoniae* is strongly influenced by nucleoid-associated proteins, we next examined the role of HU (*HlpA*), a histone-like protein known to induce DNA bending and constrain negative supercoils [33, 53]. Using an inducible HU expression system, we found that altering HU levels systematically reshaped the output of SpnD39III-regulated promoters in a manner consistent with topology-dependent control. Promoters previously identified as methylation-activated (*uraA, SPD_0554*) behaved as relaxation-favoured loci: their activity increased under HU depletion, which promotes DNA relaxation, and decreased under HU induction. Conversely, promoters repressed by methylation (*ruvB, adh*) exhibited increased activity under HU induction and reduced activity when HU was depleted. Notably, while HU modulation altered the magnitude of promoter activity, the relative ordering of methylated versus non-methylated constructs was largely preserved, indicating that methylation establishes a baseline topological state upon which HU acts as a modulator rather than a switch, and that HU does not confer methylation specificity but instead tunes transcription through broader effects on DNA topology. These observations provide a mechanistic framework that reconciles methylation-dependent activation and repression within a single model: SpnD39III methylation alters local DNA topology, shifting promoters along a spectrum of supercoiling sensitivity. Promoters intrinsically favoured by relaxed DNA are activated by methylation, whereas those preferring higher negative supercoiling are repressed. HU then tunes transcription by globally adjusting DNA topology, amplifying or dampening methylation effects depending on promoter architecture. Such behaviour mirrors classical supercoiling-sensitive promoters in bacteria and supports the idea that epigenetic regulation by Type I RM systems operates through physical properties of DNA rather than sequence-specific recruitment of transcription factors. Accordingly, epigenetic regulation via DNA topology may represent a general feature of Type I RM systems, extending beyond the pneumococcal SpnD39III system.

To then test whether this topology-mediated mechanism extends beyond pneumococcus, we examined the prototypic Type I RM system EcoKI in *E. coli*. Using a low-copy *lux* reporter plasmid, methylome sequencing, targeted mutagenesis of *hsdM* and *hsdR*, and transcriptional validation by qPCR and RNA-seq, we observed promoter-specific effects of EcoKI methylation that closely parallel those seen for SpnD39III. As in pneumococcus, EcoKI methylation could either enhance or repress promoter activity depending on context, and loss of the methyltransferase, but not the restriction subunit, abolished these effects. Importantly, RNA-seq analysis revealed widespread transcriptional changes in the *hsdM* mutant, including enrichment of CRP-regulated operons, suggesting that methylation-dependent topology changes intersect with global regulatory networks rather than acting in isolation. Notably, the promoters analysed here were selected for mechanistic investigation of methylation-dependent regulation rather than because they exhibited the largest transcriptional changes.

Importantly, these findings differ from the conclusion of Mehershahi and Chen [45], who reported little evidence for global transcriptional regulation by EcoKI. Notably, re-analysis of their publicly deposited WT MG1655 and ΔEcoKI transcriptomes confirmed that the specific genes examined here follow the same directional trends, underscoring how genome-wide analyses and locus-specific effects may yield different interpretations of the transcriptional consequences of methylation.

Together, these findings support a conserved model in which Type I RM systems function as epigenetic regulators by modulating DNA topology, thereby influencing promoter accessibility and transcriptional output. Rather than overriding canonical gene regulatory mechanisms, methylation alters the physical configuration of DNA, reshaping the regulatory landscape through which transcriptional control is exerted. This mechanism provides a parsimonious explanation for how phase-variable methylation systems can generate heritable yet reversible transcriptional programs without requiring specialized regulatory factors and may represent a general strategy by which bacteria exploit DNA modification to integrate environmental signals, genome organization, and gene regulation.

## Supplementary Material

gkag944_Supplemental_File

## Data Availability

RNA-seq data have been deposited in the NIH Gene Expression Omnibus (GEO) under accession numbers GSE293985 and GSE328962. PacBio genome assemblies are available under BioProject accession number PRJNA1416954. All GraphPad Prism data files and raw data in Excel format will be made available via the Institutional Research Repository AMSActa (https://amsacta.unibo.it/) of the University of Bologna (https://doi.org/10.6092/unibo/amsacta/9037).
