# Supplementary material for: Type I restriction–modification systems regulate gene expression through DNA topology
Source: Nucleic Acids Res. 2026 Sep 30;54(18):gkag944. doi: 10.1093/nar/gkag944 (PMC13624659; doi:10.1093/nar/gkag944)
Supplement: Supplementary file 1 [file gkag944_supplemental_file.pdf]

Type I Restriction–Modification Systems Regulate Gene Expression through DNA Topology

Althari et al.,

Supplementary materials

Table S1: Bacterial strains used in the study

Table S2: Plasmids used in the study

Table S3: Primer sequences

Table S4: SpnD39IIIA, B, C, D, E target sites in intergenic regions and downstream gene expression in SpnD39IIIA, B, and E phase-locked strains.

Table S5: PacBio-detected DNA methylation motifs in the *E. coli* MG1655 wild-type genome.

Table S6: PacBio-detected DNA methylation motifs in the *E. coli* DH5α genome.

Table S7: PacBio-detected DNA methylation motifs in the *E. coli* MG1655 Δ*hsdM* genome.

Table S8: PacBio-detected DNA methylation motifs in the *S. pneumoniae* MRO883 SpnIIIB phase-locked strain.

Table S9: PacBio-detected DNA methylation motifs in the *S. pneumoniae* MRO886 SpnIIIA phase-locked strain.

Table S10: PacBio-detected DNA methylation motifs in the *S. pneumoniae* MRO956 SpnIIIE phase-locked strain.

Table S11: PacBio-detected DNA methylation motifs in the *S. pneumoniae* MRO957 SpnIIIE phase-locked strain, carrying recombinant pDP1.

S. pneumonia synthetic DNA constructs

E. coli synthetic DNA constructs

Figure S1: Schematic maps of the luciferase plasmids and chromosomal insertion sites in *S. pneumoniae* strains.

Figure S2: Influence of EcoKI methylation on argR and rnc promoter activities across assays and EcoKI backgrounds.

Figure S3: Two-dimensional chloroquine gels of native pDP1 plasmids isolated from MRO730 (SpnD39IIIE) and MRO883 (SpnD39IIIB) D39 strains.

Table S1: Bacterial strains used in the study

Strain	Background	Relevant characteristics	Source or reference
MRO550	D39	Wildtype *S. pneumoniae* strain, enriched *SpnD39IIIE* strain, rpsL* (SPD_0251).	(10)
MRO728	D39	Δ(*hsdS-TRD1.2, -TRD2.2, -TRD2.3, creX)::add9; rpsL**	(10)
MRO729	D39	Δ(*hsdS-TRD1.2, -TRD2.1, -TRD2.3, creX)::add9; rpsL**	(10)
MRO730	D39	Δ(*hsdS-TRD1.2, -TRD2.1, -TRD2.2, creX)::add9; rpsL**	(10)
MRO750	D39	*bgaA::lucAB aphIII rpsL;* Δ(*hsdS-TRD1.2, -TRD2.2, -TRD2.3, creX)::add9; rpsL**	This study
MRO751	D39	*bgaA::lucAB aphIII rpsL;* Δ *(hsdS-TRD1.2, -TRD2.1, -TRD2.3, creX)::add9; rpsL*	This study
MRO752	D39	*bgaA::lucAB aphIII rpsL;* Δ *(hsdS-TRD1.2, -TRD2.1, -TRD2.2, creX)::add9; rpsL**	This study
MRO859	D39	*bgaA::lucAB aphIII rpsL;* Δ(*hsdS-TRD1.1, -TRD1.2, -TRD2.1, -TRD2.2, -TRD2.3, creX)::add9; rpsL**	(10)
MRO886	D39	*bgaA::*P_{uraA} *lucAB;* Δ(*hsdS-TRD1.2, -TRD2.2, -TRD2.3, creX)::add9; rpsL**	This study
MRO883	D39	*bgaA::*P_{uraA} *lucAB;* Δ(*hsdS-TRD1.2, -TRD2.1, -TRD2.3, creX)::add9; rpsL**	This study
MRO889	D39	*bgaA::*P_{uraA} *lucAB;* Δ(*hsdS-TRD1.1, -TRD1.2, -TRD2.1, -TRD2.2, -TRD2.3, creX)::add9; rpsL**	This study
MRO885	D39	*bgaA::*$P_{uraA\_T167G\ and\ T169C}$ *lucAB;*Δ(*hsdS-TRD1.2, -TRD2.2, -TRD2.3, creX)::add9; rpsL**	This study
MRO882	D39	*bgaA::*$P_{uraA\_T167G\ and\ T169C}$ *lucAB;* Δ(*hsdS-TRD1.2, -TRD2.1, -TRD2.3, creX)::add9; rpsL**	This study
MRO888	D39	*bgaA::*$P_{uraA\_T167G\ and\ T169C}$ *lucAB;*Δ(*hsdS-TRD1.1, -TRD1.2, -TRD2.1, -TRD2.2, -TRD2.3, creX)::add9; rpsL**	This study
MRO887	D39	*bgaA::*P_{uraA_T167G} *lucAB;* Δ(*hsdS-TRD1.2, -TRD2.2, -TRD2.3, creX)::add9; rpsL**	This study
MRO884	D39	*bgaA::*P_{uraA_T167G} *lucAB;* Δ(*hsdS-TRD1.2, -TRD2.1, -TRD2.3, creX)::add9; rpsL**	This study
MRO890	D39	*bgaA::*P_{uraA_T167G} *lucAB;* Δ(*hsdS-TRD1.1, -TRD1.2, -TRD2.1, -TRD2.2, -TRD2.3, creX)::add9; rpsL**	This study
MRO876	D39	*bgaA::*P_{spxB} *lucAB;*Δ(*hsdS-TRD1.2, -TRD2.2, -TRD2.3, creX)::add9; rpsL**	This study
MRO992	D39	*bgaA::*P_{spxB} *lucAB;* Δ(*hsdS-TRD1.2, -TRD2.1, -TRD2.3, creX)::add9; rpsL**	This study
MRO880	D39	*bgaA::*P_{spxB} *lucAB;* Δ(*hsdS-TRD1.1, -TRD1.2, -TRD2.1, -TRD2.2, -TRD2.3, creX)::add9; rpsL**	This study
MRO994	D39	*bgaA::*$P_{spxB\_A82C\ and\ T80G}$ *lucAB;*Δ(*hsdS-TRD1.2, -TRD2.2, -TRD2.3, creX)::add9; rpsL**	This study
MRO873	D39	*bgaA::*$P_{spxB\_A82C\ and\ T80G}$ *lucAB;* Δ(*hsdS-TRD1.2, -TRD2.1, -TRD2.3, creX)::add9; rpsL**	This study
MRO879	D39	*bgaA::*$P_{spxB\_A82C\ and\ T80G}$ *lucAB;* Δ(*hsdS-TRD1.1, -TRD1.2, -TRD2.1, -TRD2.2, -TRD2.3, creX)::add9; rpsL**	This study

MRO878	D39	*bgaA::*P$_{spxB_A82C}$ *lucAB;* Δ(*hsdS-TRD1.2, -TRD2.2, -TRD2.3, creX)::add9; rpsL**	This study
MRO875	D39	*bgaA::*P$_{spxB_A82C}$ *lucAB;* Δ(*hsdS-TRD1.2, -TRD2.1, -TRD2.3, creX)::add9; rpsL**	This study
MRO881	D39	*bgaA::*P$_{spxB_A82C}$ *lucAB;* Δ(*hsdS-TRD1.1, -TRD1.2, -TRD2.1, -TRD2.2, -TRD2.3, creX)::add9; rpsL**	This study
TH5446	ST556	Δ(*hsdS-TRD1.2, -TRD2.2, -TRD2.3, psrA); rpsL**	(20)
TH5447	ST556	Δ(*hsdS-TRD1.2, -TRD2.1, -TRD2.3, psrA); rpsL**	(20)
MRO938	D39	*bgaA::*P$_{scrA}$ *lucAB;* Δ(*hsdS-TRD1.2, -TRD2.1, -TRD2.2, creX)::add9; rpsL**	This study
MRO939	D39	*bgaA::*P$_{scrA}$ *lucAB;* Δ(*hsdS-TRD1.1, -TRD2.1, -TRD2.2, creX)::add9; rpsL**	This study
MRO940	D39	*bgaA::*P$_{scrA\ A53C\ and\ T51C}$ *lucAB;* Δ(*hsdS-TRD1.1, -TRD2.1, -TRD2.2, creX)::add9; rpsL**	This study
MRO941	D39	*bgaA::*P$_{scrA\ A53C\ and\ T51C}$ *lucAB;* Δ(*hsdS-TRD1.2, -TRD2.1, -TRD2.2, creX)::add9; rpsL**	This study
MRO942	D39	*bgaA::*P$_{adh}$ *lucAB;* Δ(*hsdS-TRD1.2, -TRD2.1, -TRD2.2, creX)::add9; rpsL**	This study
MRO943	D39	*bgaA::*P$_{adh}$ *lucAB;* Δ(*hsdS-TRD1.1, -TRD2.1, -TRD2.2, creX)::add9; rpsL**	This study
MRO944	D39	*bgaA::*P$_{adh\ A60C\ and\ G58C}$ *lucAB;* Δ(*hsdS-TRD1.1, -TRD2.1, -TRD2.2, creX)::add9; rpsL**	This study
MRO945	D39	*bgaA::*P$_{adh\ A60C\ and\ G58C}$ *lucAB;* Δ(*hsdS-TRD1.2, -TRD2.1, -TRD2.2, creX)::add9; rpsL**	This study
MRO946	D39	*bgaA::*P$_{ruvB}$ *lucAB;*Δ(*hsdS-TRD1.2, -TRD2.2, -TRD2.3, creX)::add9; rpsL**	This study
MRO947	D39	*bgaA::*P$_{ruvB\ T75G\ and\ C74A}$ *lucAB;*Δ(*hsdS-TRD1.2, -TRD2.2, -TRD2.3, creX)::add9; rpsL**	This study
MRO948	D39	*bgaA::*P$_{ruvB}$ *lucAB;* Δ(*hsdS-TRD1.2, -TRD2.1, -TRD2.3, creX)::add9; rpsL**	This study
MRO949	D39	*bgaA::*P$_{ruvB\ T75G\ and\ C74A}$ *lucAB;* Δ(*hsdS-TRD1.2, -TRD2.1, -TRD2.3, creX)::add9; rpsL**	This study
MRO950	D39	*bgaA::*P$_{SPD-0554\ T139C\ and\ T137G}$ *lucAB;* Δ(*hsdS-TRD1.2, -TRD2.1, -TRD2.3, creX)::add9; rpsL**	This study
MRO951	D39	*bgaA::*P$_{SPD-0554}$ *lucAB;* Δ(*hsdS-TRD1.2, -TRD2.1, -TRD2.3, creX)::add9; rpsL**	This study
MRO952	D39	*bgaA::*P$_{SPD-0554\ T139C\ and\ T137G}$ *lucAB;*Δ(*hsdS-TRD1.2, -TRD2.2, -TRD2.3, creX)::add9; rpsL**	This study
MRO953	D39	*bgaA::*P$_{SPD-0554}$ *lucAB;*Δ(*hsdS-TRD1.2, -TRD2.2, -TRD2.3, creX)::add9; rpsL**	This study
ΔHU pMVHU	R6	Δ*hlp*::*cat;* pMVHU (*P$_{mal}$-hlp*, *tet*)	(35)
MRO988	D39	MRO942 Δ*hlp*::*cat;* pMVHU (*P$_{mal}$-hlp*, *tet*)	This study
MRO989	D39	MRO943Δ*hlp*::*cat;* pMVHU (*P$_{mal}$-hlp*, *tet*)	This study
MRO990	D39	MRO951 Δ*hlp*::*cat;* pMVHU (*P$_{mal}$-hlp*, *tet*)	This study
MRO991	D39	MRO953 Δ*hlp*::*cat;* pMVHU (*P$_{mal}$-hlp*, *tet*)	This study
MRO993	D39	MRO992 Δ*hlp*::*cat;* pMVHU (*P$_{mal}$-hlp*, *tet*)	This study
MRO995	D39	MRO994 Δ*hlp*::*cat;* pMVHU (*P$_{mal}$-hlp*, *tet*)	This study
MRO996	D39	MRO883 Δ*hlp*::*cat;* pMVHU (*P$_{mal}$-hlp*, *tet*)	This study
MRO997	D39	MRO886 Δ*hlp*::*cat;* pMVHU (*P$_{mal}$-hlp*, *tet*)	This study
MRO998	D39	MRO938 Δ*hlp*::*cat;* pMVHU (*P$_{mal}$-hlp*, *tet*)	This study
MRO999	D39	MRO939 Δ*hlp*::*cat;* pMVHU (*P$_{mal}$-hlp*, *tet*)	This study
MRO1000	D39	MRO946 Δ*hlp*::*cat;* pMVHU (*P$_{mal}$-hlp*, *tet*)	This study
MRO1001	D39	MRO948 Δ*hlp*::*cat;* pMVHU (*P$_{mal}$-hlp*, *tet*)	This study
MG1655		Complete functional EcoKI	(56)

DH5α		*hsdR17*(r_{k}^{-} m_{k+})	(57)
SM10λ*pir*		*thi1*, *thr1*, *leuB6*, *supE44*, *tonA21*, *lacy*, *recA*::RP4-2-Tc::Mu Km^{r}	(58)
CC118		*ara*D139, Δ(*ara leu*)7697, Δ*lacX74*, Δ*phoa20*, *galE*, *galK*, *thi*, *rpsE*, *rpoB*, *argE*(Am), *recA1*, Rif^{r}	(59)
MRO963	MG1655	Δ*hsdM*	This study
MRO921	DH5α	pMRO893 (P_{argR}-*lux*)	This study
MRO922	DH5α	pMRO893 ($P_{argR}^{G/C}$-*lux*)	This study
MRO923	DH5α	pMRO893 (P_{prpR} -*lux*)	This study
MRO924	DH5α	pMRO893 ($P_{prpR}^{A/T}$ -*lux*)	This study
MRO925	DH5α	pMRO893 (P_{rnc} -*lux*)	This study
MRO926	DH5α	pMRO893 ($P_{rnc}^{G/C}$ -*lux*)	This study
MRO927	DH5α	pMRO893 ($P_{rnc}^{C/G,G/C}$ -*lux*)	This study
MRO981	MRO963	pMRO893 (P_{argR}-*lux*)	This study
MRO982	MRO963	pMRO893 ($P_{argR}^{G/C}$-*lux*)	This study
MRO983	MRO963	pMRO893 (P_{prpR} -*lux*)	This study
MRO984	MRO963	pMRO893 ($P_{prpR}^{A/T}$ -*lux*)	This study
MRO985	MRO963	pMRO893 (P_{rnc} -*lux*)	This study
MRO986	MRO963	pMRO893 ($P_{rnc}^{G/C}$ -*lux*)	This study
MRO987	MRO963	pMRO893 ($P_{rnc}^{C/G,G/C}$ -*lux*)	This study
MRO1007	DH5α	pMRO893 (P_{yccA} -*lux*)	This study
MRO1008	MRO963	pMRO893 (P_{yccA} -*lux*)	This study
MRO1009	DH5α	pMRO893 ($P_{yccA}^{C/G}$ -*lux*)	This study
MRO1010	MRO963	pMRO893 ($P_{yccA}^{C/G}$ -*lux*)	This study
MRO1011	DH5α	pMRO893 ($P_{yccA}^{A/T}$ -*lux*)	This study
MRO1012	MRO963	pMRO893 ($P_{yccA}^{A/T}$ -*lux*)	This study
MRO956	R6	Δ(*hsdS-TRD1.2, -TRD2.1, -TRD2.2, creX)::add9; rpsL**	This study
MRO957	MRO956	Δ(*hsdS-TRD1.2, -TRD2.1, -TRD2.2, creX)::add9; rpsL**, pDP1-SpnD39IIIE	This study

Table S2: Plasmids used in the study

Plasmid	Host	Relevant characteristics	Source or reference
pDP1	*S. pneumoniae*		(42)
pDP1-SpnD39IIIE	*S. pneumoniae*	Recombinant pDP1 with 9 SpnD39IIIE sites	This study
pMVHU	*E. coli – S. pneumoniae*	P_{mal}-*hlp*, *tet*	(35)
pPP2	*E. coli*	*bla*, *tetM*, *lacZ*, and *bgaA* locus homologies	(37)
pPP3	*E. coli*	*bla*, *tetM*, *luc*, and *bgaA* locus homologies	(31)
pAKlux2.1	*E. coli*	*lux* reporter plasmid	A gift from Attila Karsi (Addgene plasmid # 14081; http://n2t.net/addgene:14081; RRID: Addgene_14081)

pMRO893	*E. coli*	Deleted *lux* promoter	This study
pRDH10	*E. coli*	*sacRB*, *mobRP4*, *ori* R6K, Cm^r, Te^r	(32)

Table S3: Primer sequences

Primer Name	Sequence	Amplified target
YC1	TCAAGAATTAATTCCTTCTTAACGCCCC	*tetM*, SPD_0516, *CBRluc*,*bgaA*
YC4	CGCGAGGCAGGCTAAACCTGC	*tetM*, SPD_0516, *CBRluc*,*bgaA*
YC31	GCCAGATGATTCGATTACTGTTGTGG	*gyrB* (qPCR)
YC32	GGGCATTAACAACTGACGACCC	*gyrB* (qPCR)
YC35	GGAAGTTGCCGAAGTTGCTG	*luc* (qPCR)
YC36	GCCTTGCCTGTTTCACGATC	*luc* (qPCR)
YA17	CAAGGAGTTTGTTATGAATAAAAAATTTTTG	pDP1~500bp
YA18B	CCTACAAATGCAAAGACTTTAGCTT AGTCATTATGAATAAAGCTTGGTTATTTTG	pDP1~500bp + SpnD39IIIE tail
YA19B	GATTCAAAGACAAAGACTTTCGCTTCAAGCTGGGGAT CCGT	Km cassette + SpnD39IIIE tail
YA20B	AAGCTAAAGTCTTTGCATTTGTAGGGATACAAATTCC TCGTAGGCG	Km cassette
YA21B	CCTACAAATGCAAAGACTTTAGCTTAGTCATTATGAA TAAAGCTTGGTTATTTTG	pDP1~500bp + SpnD39IIIE tail
YA22	CCTGTAATTACAAAGAAATACAAAAATACCT	pDP1~500bp
LG069	ACACCAGAACAACTTAAAGC	Active *hsdS* PCR
LG075b	CGCCCTTCTTGTAAGAAAGAC	Active *hsdS* PCR
LG046	TATCTCCATGCTTCTGGG	Active *hsdS* PCR
LG067	CAGACCATTGATTACATTTCTGA	Active *hsdS* PCR
LG077	GAGCCACCTCTGACAAT	Active *hsdS* PCR
LG076	ACCTAAAGGATTTCTCTGG	Active *hsdS* PCR
rpsLF	ACGGGATCCGCTACCTTGAAAGTC	*rpsL* sequencing
rpsLR	TTCGGATCCGGCAGAATTTTACG	*rpsL* sequencing
YA23	ACTCATTCCTTCACCCCACC	*hsdM*
YA27	CTGCTTAATTTACGGAACTCACAATGAAC	*hsdM*
EcoKIF	TCTAGGATCCGATGATTTTTTGTTCGGC	*EcoKI*
EcoKIR	ATAGAGTCGACGATGGTTCGTCTCCTG	*EcoKI*
pAKluxF	CGAGGTCGACGGTATCGATAAGCTTGATATCGAATTC AGGC	pAklux2.1
pAKluxR	CGAGGTCGACCTGCCCGCTTTCCAGTCGGGAAAC	pAklux2.1
YA56	ACCAAAATTAATGGATTGCACTAAATCATCACC	Cloned promoter
YA57	GCCGATTCATTAATGCAGCTGGCAC	Cloned promoter
YA7	CTTCATCAAGCGGTTTCACA	*E. coli dxs* gene (qPCR)
YA8	CGAGAAACTGGCGATCCTTA	*E. coli dxs* gene (qPCR)
YA9	CTACGATACGGGAGGGCTTA	*E. coli bla* gene (qPCR)
YA10	ATAAATCTGGAGCCGGTGAG	*E. coli bla* gene (qPCR)
YA5	GTCAGGATCCGCACCTTGTCGCCTTG	*P15Aori* from pBAD30
YA6	GGTTGCTGGCGCCTATATC	*P15Aori* from pBAD30
YA7B	AGAGTCGACCTTCATCAAGCGGTTTCACA	*dxs*
YA8B	AGAGAATTCCGAGAAACTGGCGATCCTTA	*dxs*
YA50	GAGCATCATTTCACGGAGTTTG	*luxA* (qPCR)
YA51	ATAGCGGCAGTTCCTACATTC	*luxA* (qPCR)
YA52	CCATCGCTTCGTCGTCTTTA	*gyrB* (qPCR)
YA53	CTATCGTCAGATGCCGGAAAT	*gyrB* (qPCR)

Underlined primer tail sequences

Table S4: SpnD39IIIA, B, C, D, E target sites in intergenic regions and downstream gene expression in SpnD39IIIA, B, and E phase-locked strains.

	Site	Gene [a]	*SpnD39IIIA*-locked [b]	*SpnD39III B*-locked	*SpnD39IIIE* -locked	Comment on SpnD39III site position [c]
1	SpnD39IIIE	SPD_0065 *bgaC*	2	3	2	CcpA binding site
2	SpnD39IIIB	SPD_0091	659	1080	629	-35 (possibly)
3	SpnD39IIID	SPD_0117	10	10	11	not annotated
4	SpnD39IIIE	SPD_0126 *pspA*	211	268	231	Upstream
5	SpnD39IIIA	SPD_0138	4	2	4	not annotated
6	SpnD39IIIE	SPD_0169 *ribD*	42	11	31	UTR RBS
7	SpnD39IIID	SPD_0192 *rpsJ*	1242	1618	1147	-10
8	SpnD39IIIE	SPD_0208 *rplF*	1339	1747	1113	Upstream
9	SpnD39IIIE	SPD_0214 *adk*	141	59	132	UTR start site
10	SpnD39IIIC	SPD_0238 *leuS*	178	175	189	-35
11	SpnD39IIIA	SPD_0241 *ruvB*	32	52	45	-35
12	SpnD39IIIA /SpnD39III D	SPD_0248 *glmS*	200	224	178	A NagR binding site; D UTR
13	SpnD39IIIC	SPD_0254 *polC*	53	45	55	not annotated
14	SpnD39IIIB/ SpnD39IIIE	SPD_0261 *pepC*	263	489	329	Upstream
15	SpnD39IIIB	SPD_0278 PTS	0	0	0	UTR
16	SpnD39IIIE	SPD_0368 *rnhC*	52	87	61	-35
17	SpnD39IIIB/ SpnD39IIIE	SPD_0408	52	33	40	not annotated
18	SpnD39IIIE	SPD_0428 *lacE*-1	1	1	1	not annotated
19	SpnD39IIIC	SPD_0456	20	2	1	not annotated
20	SpnD39IIIA	SPD_0460 *dnaK*	244	176	182	not annotated
21	SpnD39IIIC	SPD_0478	118	104	110	UTR start site
22	SpnD39IIID	SPD_0495	6	12	7	-35
23	SpnD39IIID	SPD_0518	5	3	3	not annotated
24	SpnD39IIIC	SPD_0520	3	2	2	not annotated
25	SpnD39IIIC	SPD_0527	3	4	3	-35
26	SpnD39IIIA	SPD_0533	196	150	206	-35
27	SpnD39IIID	SPD_0550 *rplK*	1042	1647	928	-35
28	SpnD39IIIB	SPD_0554	21	38	17	Upstream
29	SpnD39IIIE	SPD_0580 *gki*	86	76	87	upstream
30	SpnD39IIIE	SPD_0592	3	6	4	-35 (possibly)

31	SpnD39IIIC	SPD_0619	8	7	6	Upstream
32	SpnD39IIIB	SPD_0623 *thiM*	35	15	32	not annotated
33	SpnD39IIIA	SPD_0625	2	2	2	Upstream
34	SpnD39IIIB	SPD_0633	2	4	2	Upstream
35	SpnD39IIIB	SPD_0636 *spxB*	1781	3026	1990	UTR start site
36	SpnD39IIID	SPD_0642	0	0	0	not annotated
37	SpnD39IIIE	SPD_0646	58	81	61	UTR
38	SpnD39IIIE	SPD_0647	16	19	23	-35
39	SpnD39IIIA /SpnD39IIIE	SPD_0650 *clpP*	206	257	239	-35
40	SpnD39IIIB	SPD_0657	12	11	11	-35; A CcpA binding site
41	SpnD39IIIA	SPD_0658 *prfB*	126	185	124	UTR
42	SpnD39IIIA	SPD_0681	25	46	24	Upstream
43	SpnD39IIIE	SPD_0717	88	147	90	Upstream
44	SpnD39IIID	SPD_0717 *clpE*	88	147	90	-10
45	SpnD39IIIB	SPD_0719	59	41	52	Upstream
46	SpnD39IIIE	SPD_0723 *rpiA*	77	100	79	Upstream
47	SpnD39IIIB	SPD_0723 *rpiA*	77	100	79	Upstream
48	SpnD39IIIB	SPD_0732 *rpsT*	400	566	396	-10
49	SpnD39IIIE	SPD_0735	27	43	29	Upstream
50	SpnD39IIIE	SPD_0762 *sufCDS*	531	586	574	Upstream
51	SpnD39IIIA	SPD_0767 *dacC*	64	69	69	Upstream
52	SpnD39IIIA	SPD_0768	67	66	64	not annotated
53	SpnD39IIIB	SPD_0775	23	47	24	Upstream
54	SpnD39IIID	SPD_0785	104	67	99	not annotated
55	SpnD39IIIA	SPD_0787 *pepX*	7	6	6	Upstream
56	SpnD39IIIA	SPD_0803	77	58	78	Upstream
57	SpnD39IIIB	SPD_0804	4	6	6	not annotated
58	SpnD39IIIE	SPD_0811 *speE*	57	36	40	not annotated
59	SpnD39IIIC	SPD_0822	56	43	61	UTR
60	SpnD39IIIE	SPD_0839	143	193	137	not annotated
61	SpnD39IIIC	SPD_0847 *infC*	550	548	463	UTR
61	SpnD39IIIB	SPD_0847 *infC*	550	548	463	Upstream
63	SpnD39IIIB	SPD_0854 *pavA*	40	50	43	Upstream
64	SpnD39IIIC	SPD_0855	79	84	77	not annotated
65	SpnD39IIIB	SPD_0873	58	64	58	not annotated
66	SpnD39IIID	SPD_0882	5	5	4	not annotated
67	SpnD39IIIA	SPD_0882	5	5	4	not annotated
68	SpnD39IIIB	SPD_0894 *pepT*	128	179	134	Upstream

69	SpnD39IIIC	SPD_0901<br>*dapA*	43	27	44	not annotated
70	SpnD39IIIA	SPD_0939	8	8	6	not annotated
71	SpnD39IIIC	SPD_0973<br>*pcrA*	57	54	55	Upstream
72	SpnD39IIIB	SPD_1010	16	18	18	not annotated
73	SpnD39IIIB	SPD_1019	62	66	63	Upstream
74	SpnD39IIID	SPD_1022	0	0	0	not annotated
75	SpnD39IIIE	SPD_1049<br>*lacT*	0	0	0	not annotated
76	SpnD39IIIC	SPD_1069	65	78	60	not annotated
77	SpnD39IIIE	SPD_1079	37	24	34	-35
78	SpnD39IIIC	SPD_1107<br>*guaC*	59	79	38	UTR
79	SpnD39IIIC	SPD_1109	5	4	5	not annotated
80	SpnD39IIIE	SPD_1120<br>*topA*	135	145	124	UTR
81	SpnD39IIIB	SPD_1121	41	23	38	UTR
82	SpnD39IIIB	SPD_1139<br>*lemA*	142	223	157	not annotated
83	SpnD39IIIB	SPD_1141<br>*uraA*	189	1231	100	-35; -10
84	SpnD39IIIE	SPD_1144	19	21	23	Upstream
85	SpnD39IIIB	SPD_1188<br>*rplJ*	1451	1612	1341	not annotated
86	SpnD39IIIB	SPD_1201<br>*licD3*	71	77	71	Upstream
87	SpnD39IIIB	SPD_1250<br>*nadE*	49	66	54	-10
88	SpnD39IIID	SPD_1251<br>*pncB*	48	64	55	-10
89	SpnD39IIIE	SPD_1255	3	5	3	not annotated
90	SpnD39IIIE	SPD_1274<br>*guaA*	365	471	337	UTR
91	SpnD39IIIA	SPD_1290	482	464	513	UTR
92	SpnD39IIIA	SPD_1293	331	537	374	Upstream
93	SpnD39IIIB	SPD_1299	0	0	0	Upstream
94	SpnD39IIID	SPD_1305<br>*glyQ*	81	100	79	not annotated
95	SpnD39IIIC	SPD_1310	49	87	49	not annotated
96	SpnD39IIIB	SPD_1319	5	1	5	not annotated
97	SpnD39IIIA	SPD_1326<br>*pgm*	291	247	322	not annotated
98	SpnD39IIIB	SPD_1332	62	60	68	not annotated
99	SpnD39IIIB	SPD_1354	10	4	8	not annotated
100	SpnD39IIIE	SPD_1359	47	41	48	not annotated
101	SpnD39IIIB	SPD_1367	109	120	110	not annotated
102	SpnD39IIIC	SPD_1377	1	1	1	not annotated
103	SpnD39IIIA	SPD_1437	39	57	42	Upstream
104	SpnD39IIIA	SPD_1438	217	249	209	Upstream
105	SpnD39IIIA	SPD_1450	45	70	51	Upstream
106	SpnD39IIIB	SPD_1494	18	10	15	not annotated
107	SpnD39IIIB	SPD_1506	31	27	31	not annotated
108	SpnD39IIIE	SPD_1524	78	149	85	-35
109	SpnD39IIIB	SPD_1530	46	66	50	not annotated

110	SpnD39IIIE	SPD_1532	164	207	131	A CcpA binding site
111	SpnD39IIIB	SPD_1549	240	253	240	not annotated
112	SpnD39IIIE	SPD_1568	0	2	0	not annotated
113	SpnD39IIIE	SPD_1570	42	29	39	not annotated
114	SpnD39IIIB/ SpnD39IIIE	SPD_1573 *prmA*	48	34	53	not annotated
115	SpnD39IIIB	SPD_1578	1	1	1	not annotated
116	SpnD39IIIA	SPD_1588	196	260	279	-35
117	SpnD39IIIB	SPD_1610	3	3	2	-35
118	SpnD39IIIA	SPD_1635 *galR*	7	5	7	not annotated
119	SpnD39IIIA	SPD_1639	9	15	9	not annotated
120	SpnD39IIIE	SPD_1664	17	8	7	not annotated
121	SpnD39IIIB	SPD_1671 *amiA*	305	266	299	Upstream
122	SpnD39IIIA	SPD_1678 *aga*	14	26	14	not annotated
123	SpnD39IIIE	SPD_1736	2	3	1	not annotated
124	SpnD39IIIB	SPD_1744	2	1	2	not annotated
125	SpnD39IIIE	SPD_1770	0	0	0	not annotated
126	SpnD39IIIA	SPD_1772	11	14	10	-35
127	SpnD39IIIA	SPD_1776 *purR*	231	205	224	not annotated
128	SpnD39IIIB	SPD_1782 *ksgA*	55	64	66	Upstream
129	SpnD39IIIA	SPD_1785	18	14	17	Upstream
130	SpnD39IIID	SPD_1792	7	8	6	not annotated
131	SpnD39IIIE	SPD_1796	26	26	35	Upstream
132	SpnD39IIIA	SPD_1834	35	130	71	Upstream
133	SpnD39IIIB	SPD_1840	0	1	0	A UlaR binding site
134	SpnD39IIIC	SPD_1863 *cglA*	4	1	4	A ComX binding site
135	SpnD39IIIB	SPD_1869	26	35	32	not annotated
136	SpnD39IIIE	SPD_1874	24	25	22	not annotated
137	SpnD39IIIB	SPD_1905 *argS*	146	214	199	not annotated
138	SpnD39IIIE	SPD_1910 *pstS*	5	2	5	not annotated
139	SpnD39IIIB	SPD_1925 *pbplB*	58	76	65	-35; -10
140	SpnD39IIIB	SPD_1926 *tyrS*	336	594	382	not annotated
141	SpnD39IIIE	SPD_1935 *malC*	15	12	13	not annotated
142	SpnD39IIIA	SPD_1954	11	9	11	not annotated
143	SpnD39IIIC	SPD_1961	4	5	4	not annotated
144	SpnD39IIIA	SPD_1982	6	3	6	not annotated
145	SpnD39IIIE	SPD_2013 *glpK*	1	3	1	A CcpA binding site
146	SpnD39IIIB	SPD_2027	30	15	28	UTR
147	SpnD39IIIE	SPD_2027	30	15	28	UTR
148	SpnD39IIIB	SPD_2052	57	39	56	not annotated

[a] Gene annotation in D39 (GenBank accession: CP000410).

[b] raw normalised sequence reads. The grey shaded RNAseq data are those which have methylated promoter region (host specificity match the intergenic SpnD39III site).

[c] promoter and regulatory sites annotation as per PneumoBrowse (https://veeninglab.com/pneumobrowse).

Table S5: PacBio-detected DNA methylation motifs in the *E. coli* MG1655 wild-type genome.

Enzyme	Motif string [1]	Modification type	Fraction [2]	Detected sites	Sites in Genome	Mean score [3]
Dam	GATC	m6A	0.99	38,226	38246	432.2804
EcoKI	AACN$_6$GTGC	m6A	1	595	595	367.0521
EcoKI	GCACN$_6$GTT	m6A	1	595	595	343.7345
Dcm	CCTGG	m4C	0.53	2089	3937	78.59837

1 N: A/T/C/G, R: A/G, Y: C/T, V: G/A/C, H: A/T/C, D: G/A/T, W: A/T.
2 Fraction: proportion of relevant motif in the whole genome.
3 Mean score: to evaluate the strength of modified base signal directly.

Table S6: PacBio-detected DNA methylation motifs in the *E. coli* DH5α genome.

Enzyme	Motif string [1]	Modification type	Fraction [2]	Detected sites	Sites in Genome	Mean score [3]
Dam	GATC	m6A	0.99	37771	37784	467.509
EcoKI	AACN$_6$GTGC	m6A	1	588	588	395.659
EcoKI	GCACN$_6$GTT	m6A	1	588	588	372.483
Dcm	CCTGG	m4C	0.54	2109	3892	81.6472

1 N: A/T/C/G, R: A/G, Y: C/T, V: G/A/C, H: A/T/C, D: G/A/T, W: A/T.
2 Fraction: proportion of relevant motif in the whole genome.
3 Mean score: to evaluate the strength of modified base signal directly.

Table S7: PacBio-detected DNA methylation motifs in the *E. coli* MG1655 Δ*hsdM* genome.

Enzyme	Motif string [1]	Modification type	Fraction [2]	Detected sites	Sites in Genome	Mean score [3]
Dam	GATC	m6A	0.99	38230	38240	411.9085
Dcm	CCTGG	m4C	0.546	2150	3937	78.76325

1 N: A/T/C/G, R: A/G, Y: C/T, V: G/A/C, H: A/T/C, D: G/A/T, W: A/T.
2 Fraction: proportion of relevant motif in the whole genome.
3 Mean score: to evaluate the strength of modified base signal directly.

Table S8: PacBio-detected DNA methylation motifs in the *S. pneumoniae* MRO883 SpnIIIB phase-locked strain.

Enzyme	Motif string [1]	Modification type	Fraction [2]	Detected sites	Sites in Genome	Mean score [3]
SpnD39II	TCGAG	m6A	0.997365	1514	1518	471.6004
SpnD39III-B	GAANNNNNNNNNTTYG	m6A	1	1042	1042	515.3388
SpnD39III-B	CRAANNNNNNNNNTTC	m6A	1	1042	1042	492.1296
SpnD39I	TCTAGA	m6A	1	648	648	528.213

1 N: A/T/C/G, R: A/G, Y: C/T, V: G/A/C, H: A/T/C, D: G/A/T, W: A/T.
2 Fraction: proportion of relevant motif in the whole genome.
3 Mean score: to evaluate the strength of modified base signal directly.

Table S9: PacBio-detected DNA methylation motifs in the *S. pneumoniae* MRO886 SpnIIIA phase-locked strain.

Enzyme	Motif string [1]	Modification type	Fraction [2]	Detected sites	Sites in Genome	Mean score [3]
SpnD39II	TCGAG	m6A	0.99668	1501	1506	516.8521
SpnD39III -A	CAGNNNNNNNNTTYG	m6A	1	718	718	593.5362
SpnD39III -A	CRAANNNNNNNNCTG	m6A	1	718	718	531.9569
SpnD39I	TCTAGA	m6A	1	648	648	573.5216

1 N: A/T/C/G, R: A/G, Y: C/T, V: G/A/C, H: A/T/C, D: G/A/T, W: A/T.
2 Fraction: proportion of relevant motif in the whole genome.
3 Mean score: to evaluate the strength of modified base signal directly.

Table S10: PacBio-detected DNA methylation motifs in the *S. pneumoniae* MRO956 SpnIIIE phase-locked strain.

Enzyme	Motif string [1]	Modification type	Fraction [2]	Detected sites	Sites in Genome	Mean score [3]
SpnD39II	TCGAG	m6A	0.996687	1504	1509	464.1336
SpnD39III -E	AAGNNNNNNNNTTYG	m6A	1	1024	1024	533.4785
SpnD39III -E	CRAANNNNNNNNCTT	m6A	1	1024	1024	484.8897
SpnD39I	TCTAGA	m6A	1	642	642	516.0421

1 N: A/T/C/G, R: A/G, Y: C/T, V: G/A/C, H: A/T/C, D: G/A/T, W: A/T.
2 Fraction: proportion of relevant motif in the whole genome.
3 Mean score: to evaluate the strength of modified base signal directly.

Table S11: PacBio-detected DNA methylation motifs in the *S. pneumoniae* MRO957 SpnIIIE phase-locked strain, carrying recombinant pDP1.

Enzyme	Motif string [1]	Modification type	Fraction [2]	Detected sites	Sites in Genome	Mean score [3]
SpnD39II	TCGAG	m6A	0.9967	1510	1515	474.6464
SpnD39III-E	AAGNNNNNNNNTTYG	m6A	1	1033	1033	540.4647
SpnD39III-E	CRAANNNNNNNNCTT	m6A	1	1033	1033	492.7948
SpnD39I	TCTAGA	m6A	1	642	642	523.5249

1 N: A/T/C/G, R: A/G, Y: C/T, V: G/A/C, H: A/T/C, D: G/A/T, W: A/T.
2 Fraction: proportion of relevant motif in the whole genome.
3 Mean score: to evaluate the strength of modified base signal directly.

***S. pneumonia* synthetic DNA constructs**

The synthetic DNA constructs (ThermoFisher Scientific, UK) contained two regions of homology to the chromosomal locus engineered for expression (500bp upstream and 500 bp downstream); to allow for integration during transformation. Intergenic regions of *spxB* SPD_0636 (190 bp), *uraA* SPD_1141 (206 bp), SPD_0554 (254 bp), *ruvB* SPD_0241 (123 bp), *adh* SPD_1865 (138 bp), and *scrA* SPD_1532 (130 bp). Labelling is as follows (SpnD39III site yellow highlight; green bold, -35 and -10 promoter consensus; underlined 5'UTR; bold blue, start codon; cyan highlight, TSS; bold black, RBS; red bold, mutation introduced).

>upstream homology

TAATTTTCATGTGATTTTCCTCCATTCAAAAGCCCAAAAGGGCATAAAAATCCCAGTGATAA
ATACTCTTATCACTGGGATTTTTATGCATAACCATAGGCATACAAAGCATACAGATATTCTC
CGGATACTTTAGAATCACATGATAAAGGTATTCTTAAACTGGGTACAAAAAACTAAGCCCTC
CTAAAAAAGGACATCCAATTATTTGTTCCCGCTATCAAATTGACAGTTTATTTAAGAATACC
TTGCCGCATATTTATTAACTCCTTTTAAATAGATACTTAAATTATAGCACGTAAGAGCATAT
TTGTAAAGGAATCTCCAATTTTTTATCAAAAAGAGTACATGATTACAAAGTATCTGTAATCA
TGTACCAATATTTGTTATTTTACAATCTTCCAATTATCTTGTTTTTCTAATATCAACTCAAA
TTGCGAGATTTGGGTTGCCTTTGTTTCTTGATCTAAATATTTCACAGCGACATTCACGATTA
CTTG

>downstream homology

GTCAAGCGGGAAAAAAACGTTATTTACGGTCCAGAACCCCTCCACCCGCTAGAAGACTTAAC
GGCTGGAGAAATGTTATTCCGTGCCCTCCGTAAGCACAGCCACTTACCACAGGCCTTAGTTG
ATGTAGTTGGTGACGAAAGCTTGTCATATAAAGAATTCTTTGAAGCTACAGTCCTTTTGGCA
CAATCATTGCACAACTGTGGGTATAAGATGAATGACGTCGTTTCAATTTGTGCAGAAAACAA
TACCCGTTTTTTCATCCCAGTTATTGCTGCTTGGTACATCGGCATGATTGTCGCTCCTGTAA
ACGAATCATATATCCCAGATGAATTATGCAAGGTCATGGGAATCTCTAAGCCTCAAATTGTT
TTTACGACTAAGAACATTTTGAACAAGGTTTTAGAAGTGCAAAGCCGCACCAACTTCATTAA
ACGGATCATTATTTTGGACACCGTTGAAAACATTCATGGGTGTGAATCATTACCAAACTTCA
T

>*spxB* SPD0636 WT (SpnD39IIIB)

TCTTTGTTATAAAACAGAAATGACAACACTTTCAAAACTGTATTAGAATAGAGAATAGAGAG
TTTTGAGCAGATTTT**TAGAAA**AGTCAGCATAAA**TATGATACAGT**GGAATAGTAAAAATTTGG
AGAACGTTTCCAATTCTATGTAATCGTATTCTCCAAGTTTAAAAAAATTGA**AGGAGA**GTTAT
CATT**ATG**

>*spxB* SPD0636 SpnD39IIIA

TCTTTGTTATAAAACAGAAATGACAACACTTTCAAAACTGTATTAGAATAGAGAATAGAGAG
TTTTGAGCAGATTTT**TAGAAA**AGTCAGCATAAA**TATGATACAGT**GG**C**A**G**AGTAAAAATTTGG
AGAACGTTTCCAATTCTATGTAATCGTATTCTCCAAGTTTAAAAAAATTGA**AGGAGA**GTTAT
CATT**ATG**

>*spxB* SPD0636 no-SpnD39III

TCTTTGTTATAAAACAGAAATGACAACACTTTCAAAACTGTATTAGAATAGAGAATAGAGAG
TTTTGAGCAGATTTT**TAGAAA**AGTCAGCATAAA**TATGATACAGT**GG**C**ATAGTAAAAATTTGG
AGAACGTTTCCAATTCTATGTAATCGTATTCTCCAAGTTTAAAAAAATTGA**AGGAGA**GTTAT
CATT**ATG**

>*uraA* SPD1141 WT (SpnD39IIIB)

TCTAGGCTCGGGAAAAAATGA**TTTACA**AAATCATTTTTTTC**TGCTATACT**ATCCTAAGCAAA
GGTTTTTAATGTCATCCCGTGAGGTGACGAAGACGCAAAAATATTTGAAACTCTTTAAAGTC
TAGATTTTAGAGAAGTCTTACTCTGAGGGCCTATTGCTGTAAAATAATGGGCTCTTTTCTGA
TGCCCAAAAGTGAGGTTTAT**ATG**

>*uraA* SPD1141 SpnD39IIIA

TCTAGGCTCGGGAAAAAATGA**TTTACA**AAATCATTTT**C**T**G**C**TGCTATACT**ATCCTAAGCAAA
GGTTTTTAATGTCATCCCGTGAGGTGACGAAGACGCAAAAATATTTGAAACTCTTTAAAGTC
TAGATTTTAGAGAAGTCTTACTCTGAGGGCCTATTGCTGTAAAATAATGGGCTCTTTTCTGA
TGCCCAAAAGTGAGGTTTAT**ATG**

>*uraA* SPD1141 no-SpnD39III

TCTAGGCTCGGGAAAAAATGA**TTTACA**AAATCATTTTTT**G**C**TGCTATACT**ATCCTAAGCAAA
GGTTTTTAATGTCATCCCGTGAGGTGACGAAGACGCAAAAATATTTGAAACTCTTTAAAGTC
TAGATTTTAGAGAAGTCTTACTCTGAGGGCCTATTGCTGTAAAATAATGGGCTCTTTTCTGA
TGCCCAAAAGTGAGGTTTAT**ATG**

>SPD_0554 WT (SpnD39IIIB)

TGGTGAGAAGATGAGTAGGTTGGTAATTTAAACTATTAAACAGAATTTTTGATTAAAAGTAT
TATTTTATGAGAGAAATCCTAATTTCACAATCCATAGGCAAACTCTTGCATTTCGTTTTTTA
TTGGAC**TATAAT**AGGTTGGTATAAAGCCTTTTGTAGTAATAAAATGTAGAAGGTGTAGAAAG

CAAGGATTTAGAATATTTGTAGTTAAAAACACAATGTTGCTATTCCTTACGATA**GGGAGA**TA
GAT**ATG**

>SPD_0554 SpnD39IIIA

TGGTGAGAAGATGAGTAGGTTGGTAATTTAAACTATTAAACAGAATTTTTGATTAAAAGTAT
TATTTTATGAGAGAAATCCTAATTTCACAATCCATAGGCAAACTCTTGCA**CTG**CGTTTTTTA
TTGGAC**TATAAT**AGGTTGGTATAAAGCCTTTTGTAGTAATAAAATGTAGAAGGTGTAGAAAG
CAAGGATTTAGAATATTTGTAGTTAAAAACACAATGTTGCTATTCCTTACGATA**GGGAGA**TA
GAT**ATG**

>*adh* SPD1865 WT (SpnD39IIIE)

TTCTGCTTTTTTATGCAGGGTACTTTACTGGTAAATAAAAAGTTAAAAAAATCACAAAAAAA
GC**TTGAAG**AAACAAAGAAAAAGGC**TTACAAT**TGAACTATAAATATTACAAATAAAAAAACGG
AGGAGTGCTTT**ATG**

>*adh* SPD1865 SpnD39IIIF

TTCTGCTTTTTTATGCAGGGTACTTTACTGGTAAATAAAAAGTTAAAAAAATCACAAAAAAA
GC**TTGAAG**AAACA**C**A**C**AAAAAGGC**TTACAAT**TGAACTATAAATATTACAAATAAAAAAACGG
AGGAGTGCTTT**ATG**

>*ruvB* SPD_0241 WT (SpnD39IIIA)

GTATAATATTATACCACATAAGTGCTAAACTTAGGAGTCACCCAATCAGGTGGCTTTT**TTGT**
TTGTGGCTTGGTTT**TTTGATATAAT**AGAGCCATGAGTAGAATTTTAGATAATGAGATA**ATG**

>*ruvB* SPD_0241 SpnD39IIIB

GTATAATATTATACCACATAAGTGCTAAACTTAGGAGTCACCCAA**GA**AGGTGGCTTTT**TTGT**
TTGTGGCTTGGTTT**TTTGATATAAT**AGAGCCATGAGTAGAATTTTAGATAATGAGATA**ATG**

>*scrA* SPD_1532 WT (SpnD39IIIE)

AGTGGATAGAATATTTTTTCTGCAAAACGC**TTGACA**TATTTTTAAAA**CATGATAAAAT**AATA
GTCGTAAGGGCGAATAAAATCGCTTTCAAACAAGAACAAAATGTTATATA**AGGAGA**TTTTTG
CAA**ATG**

>*scrA* SPD_1532 SpnD39IIIF

AGTGGATAGAATATTTTTTTCTGCAAAACGC**TTGACA**TATTTTTAAAA**CATGATAAAAT**AATA
GTCGTAAGGGCG**C**A**C**AAAATCGCTTTCAAACAAGAACAAAATGTTATATA**AGGAGA**TTTTTG
CAA**ATG**

***E. coli* synthetic DNA constructs**

In *E. coli*, the synthetic DNA constructs (ThermoFisher Scientific, UK) contained approximately 200bp of the intergenic region of the gene of interest, along with tails incorporating HindIII or SalI on one side and EcoRI cloning sites on the other to facilitate ligation into the reporter plasmid. Labelling is as follows: yellow highlight, EcoKI recognition site; cyan highlight, transcription start site (TSS); green bold, -35 and -10 promoter consensus sequences; underlined, Dam or Dcm target sequence; red bold, point mutations; black bold, HindIII, SalI and EcoRI cloning sites.

>*argR* WT

CATCCT**AAGCTT**ACTGCGACTTTCATCCTAAACTCCTTATTATATTGATAAACTAAGATATG
TTGCTCCGCTGCCGCGACCTTAATCCACAAAATTGCCGTTTACAATGACCACATCTCAAGAA
TGTGTAGTCACGCAAGTTTAGCGTTTATGCATTTAATTGCCGTAATCAGGAACCTAACTTAC
GTAATTAACAGTCGCTTATGCATTAGCGCAACATTCCAACAGGTGGTGACAATATACCCTAC
CGTTCAGCCAAAACAACATCAATTTGATAACAATTAATTTACTTTTAAGCAGAATTTGCATG
CCGTGACGCAGGCATGTTTCTCAATAACGAAATTTGATAAAATCCCGCTCTTTCATAACATT
ATTTCAGCCTTCTTCAGGGCTGACTGT**TTGCAT**AAAAATTCATCTGTATGCAC**AATAAT**GTT
GTATCAAC**GAATTC**TGTTAT

>*argR* EcoKI-

CATCCT**AAGCTT**ACTGCGACTTTCATCCTAAACTCCTTATTATATTGATAAACTAAGATATG
TTGCTCCGCTGCCGCGACCTTAATCCACAAAATTGCCGTTTACAATGACCACATCTCAAGAA
TGTGTAGTCACGCAAGTTTAGCGTTTATGCATTTAATTGCCGTAATCAGGAACCTAACTTAC
GTAATTAACAGTCGCTTATGCATTAGCGCAACATTCCAACAGGTGGTGACAATATACCCTAC
CGTTCAGCCAAAACAACATCAATTTGATAACAATTAATTTACTTTTAAGCAGAATTTGCATG
CCGTGACGCAGGCATGTTTCTCAATAACGAAATTTGATAAAATCCCGCTCTTTCATAACATT
ATTTCAGCCTTCTTCAGGGCTGACTGT**TTGCAT**AAAAATTCATCTGTATGCAC**AATAAT****C**TT
GTATCAAC**GAATTC**TGTTAT

>prpR WT

CATCCT**AAGCTT**AGCCCATCCTTTGTTATCAACTTGTTATTTGCGTTGATAAAGACAAAGCA
AGGGGTGTGCCACAATTGCCTTAATAAAATTAAATGTTTAATTTCAATTTGTTAGAGATAAA
GTCATGAACTAATCCGCAAATATGCGTTTCAGTTAACGTTTCAGGCAATGTTTCACCGCGT**T**
TCATTGCAACAATTATGAAAC**AAGACT**AAACCCAATATTCGGTTTCTTAACTTTGCGGTGCG
CT**GAATTC**TGTTAT

>*prpR* EcoKI-

CATCCT**AAGCTT**AGCCCATCCTTTGTTATCAACTTGTTATTTGCGTTGATAAAGACAAAGCA
AGGGGTGTGCCACAATTGCCTTAATAAAATTAAATGTTTAATTTCAATTTGTTAGAGATAAA
GTCATGAACTAATCCGCAAATATGCGTTTCAGTTAACGTTTCAGGCAATGTTTCACCGCGTT**T**
TCATTGCAACAATTATGAAAC**AAGACT**AAACCCAATATTCGGTTTCTTA**T**CTTTGCGGTGCG
CT**GAATTC**TGTTAT

>*rnc* WT

CATCCT**AAGCTT**TAGCCATCTTCGTTCACGTTGTCGCCGTTATGGCGACAACGTGAATTATT
TATGAGATAAATCTCCCGTGG**CTAACG**ACATCCCCCGTCGTTGTGTA**TAGAAT**ATTCCCCCG
AAGTTTAAGGTTGGCACCTCCAGGTTGCCACGGCACACGAAACAGCGTTGGTCCCCATATAC
CGGTAAACTGAAACTGCAGCGAAGCAGTTAGCAGAACCATGTATATCAGGTCTGTTTCGTGT
GCTGAATTGTTGACGCATTTATTTATTGGTATCGC**GAATTC**TGTTAT

>*rnc* EcoKI-

CATCCT**AAGCTT**TAGCCATCTTCGTTCACGTTGTCGCCGTTATGGCGACAACGTGAATTATT
TATGAGATAAATCTCCCGTGG**CTAACG**ACATCCCCCGTCGTTGTGTA**TAGAAT**ATTCCCCCG
AAGTTTAAGGTTG**C**CACCTCCAGGTTGGCACGGCACACGAAACAGCGTTGGTCCCCATATAC
CGGTAAACTGAAACTGCAGCGAAGCAGTTAGCAGAACCATGTATATCAGGTCTGTTTCGTGT
GCTGAATTGTTGACGCATTTATTTATTGGTATCGC**GAATTC**TGTTAT

>*rnc* EcoKI- Dcm-

CATCCT**AAGCTT**TAGCCATCTTCGTTCACGTTGTCGCCGTTATGGCGACAACGTGAATTATT
TATGAGATAAATCTCCCGTGG**CTAACG**ACATCCCCCGTCGTTGTGTA**TAGAAT**ATTCCCCCG
AAGTTTAAGGTTGGCA**G**CTCCAG**C**TTGCCACGGCACACGAAACAGCGTTGGTCCCCATATAC
CGGTAAACTGAAACTGCAGCGAAGCAGTTAGCAGAACCATGTATATCAGGTCTGTTTCGTGT
GCTGAATTGTTGACGCATTTATTTATTGGTATCGC**GAATTC**TGTTAT

>*yccA* WT

CATCCT**GTCGAC**AGAGTTCGAATCTCTGCGCTTCCGCCAAATAAGATAAGGGGTTAGCTAAA
TGCTAACCCCTTTTTCTTTTGCCTGTCGAAATTCTCAGGGCGTTATATTTGCTTAATGACCT
GATAATCCGCTGTTAAACCTGTTCCAGATCAAATGCGTAAAGATGGGTAAAACTTCTGGGTG
CCCTTACGCATTATCAT**TATGCT**GCTTAATTAATTACATCTGTCATAGAGAGTGACTCA**GAA**
TTCTGTTAT

>*yccA* EcoKI-

CATCCT**GTCGAC**AGAGTTCGAATCTCTGCGCTTCCGCCAAATAAGATAAGGGGTTAGCTAAA
TGCTAACCCCTTTTTCTTTTGCCTGTCGAAATTCTCAGGGCGTTATATTTGCTTAATGACCT
GATAATCCGCTGTTAAACCTGTTCCAGATCAAATGCGTAAAGATGGGTAAAACTTCTGGGTG

GCCTTACGCATTATCAT**TATGCTG**CTTAATTAATTACATCTGTCATAGAGAGTGACTCA**GAA**
TTCTGTTAT

>*yccA* Dam-

CATCCT**GTCGAC**AGAG**T**TCGAATCTCTGCGCTTCCGCCAAATAAGATAAGGGGTTAGCTAAA
TGCTAACCCCTTTTTCTTTTGCCTGTCGAAATTCTCAGGGCGTTATATTTGCTTAATGACCT
GATAATCCGCTGTTAAACCTGTTCCAGTTCAAATGCGTAAAGATGGGTAAAACTTCTGGGTG
CCCTTACGCATTATCAT**TATGCT**GCTTAATTAATTACATCTGTCATAGAGAGTGACTCA**GAA**
TTCTGTTAT

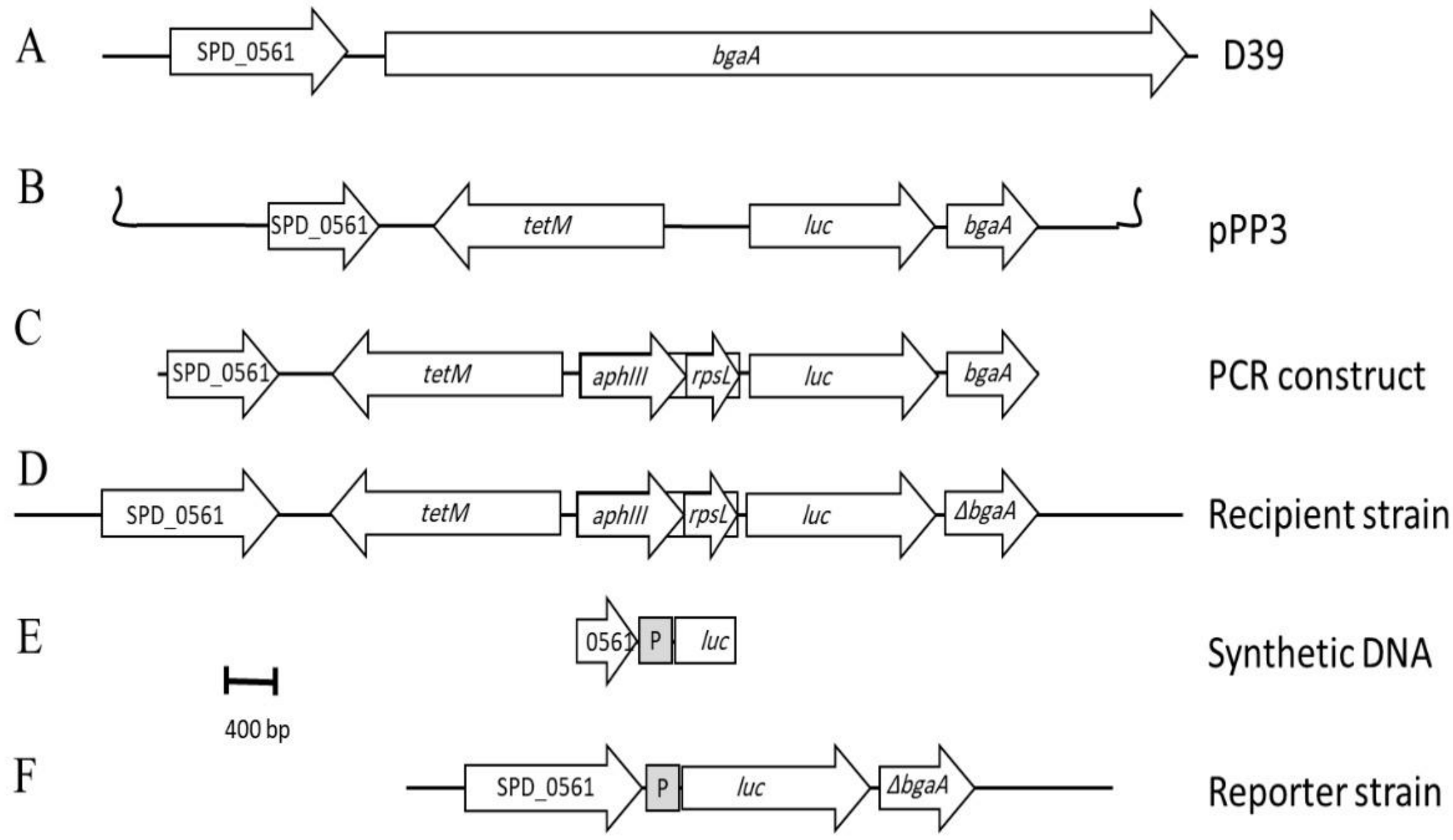

Figure S1: Schematic maps of the luciferase plasmids and chromosomal insertion sites in *S. pneumoniae* strains. (A) The map of the SPD_0561 gene and *bgaA* SPD-0562 gene in D39 *S. pneumoniae* (CP000410.2). (B) The map of the pPP3 plasmid (8793 bp), the map shows a first region of homology to the pneumococcal chromosome (part of SPD_0561; position 578,426-579,358 CP000410.2), the tetracycline resistance gene *tet*(M), a codon adjusted click beetle red-emission variant luciferases *luc* gene (1629 bp), and a further region of homology to the pneumococcal chromosome (part of *bgaA* position 585,524-586,286 (63). (C) The map of the PCR construct (7845 bp) including part of SPD_0561 (927 bp), *tet*(M), the Janus cassette for negative selection containing *aphIII* (882 bp) and *rpsL* (448 bp) (29), the *luc* gene and part of *bgaA* (761 bp). (D) The map of the locus in the recipient strain which includes between SPD_0561 and a partial version of *bgaA*, the *tet*(M) gene, the Janus cassette and *luc* (1184 bp). (E) The synthetic promoter DNA constructs containing part of SPD_0561 (500 bp), the promoter regions and part of *luc* (500 bp). (F) The map of the locus of the reporter strains containing the recombinant promoters in front of the *luc* gene.

Alt text: Schematic maps show the luciferase reporter plasmids and chromosomal insertion sites used in *S. pneumoniae*. The constructs include the pPP3 plasmid, a PCR integration construct containing *tet*(M), the Janus cassette and *luc*, the corresponding chromosomal reporter locus, and synthetic promoter constructs. The final maps show integration of recombinant promoters upstream of the *luc* reporter gene in pneumococcal strains.

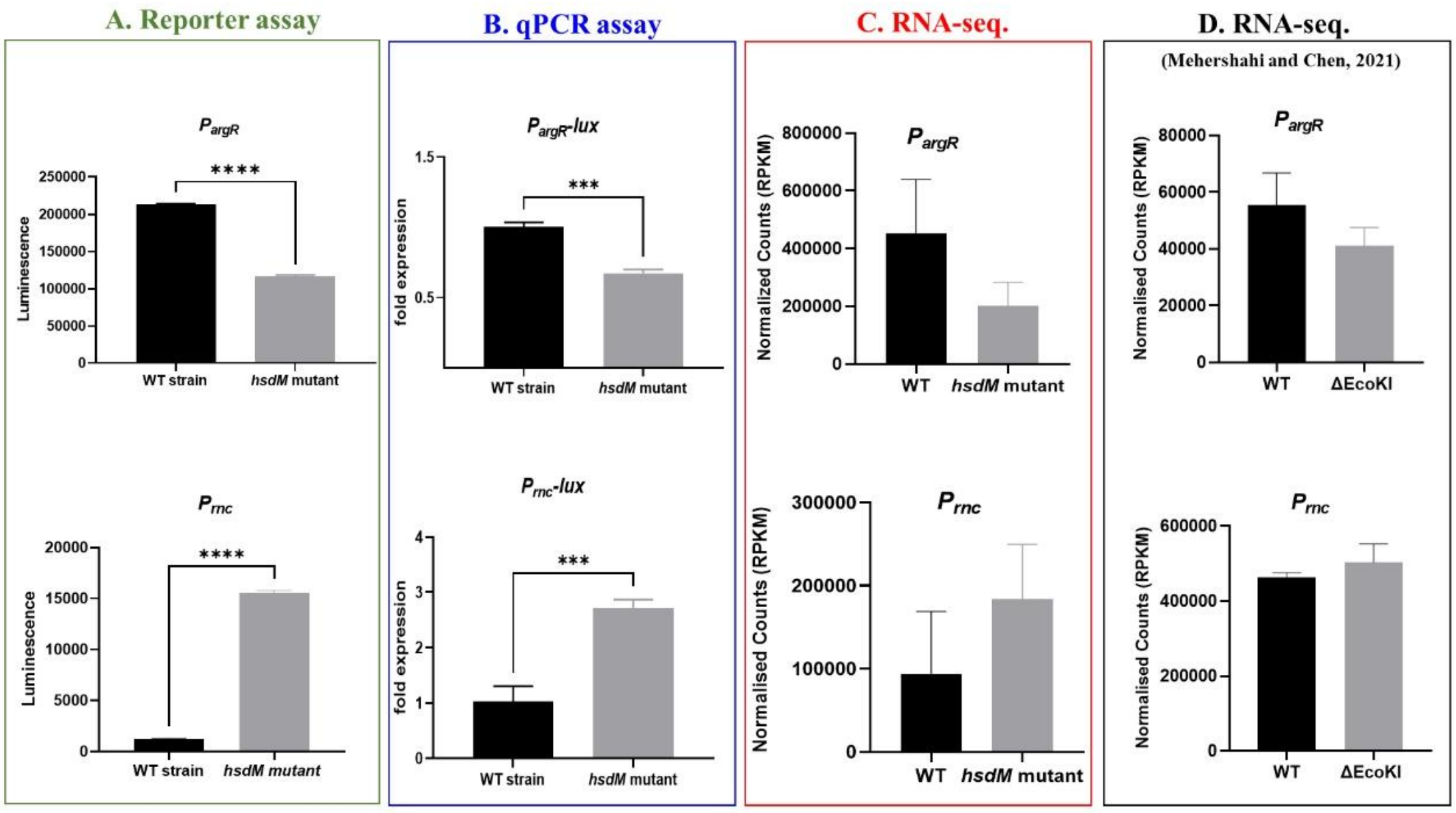

Figure S2: Influence of EcoKI methylation on *argR* and *rnc* promoter activities across assays and EcoKI backgrounds. (A) Results from a *lux* reporter assay measuring luminescence, serving as a proxy for gene expression, under the control of *argR* (top) and *rnc* (bottom) in methylation-active and passive hosts. (B) Results from a qPCR assay measuring *lux* gene transcription under the control of *argR* (top) and *rnc* (bottom) in methylation-active and passive hosts. (C) Normalised gene expression values calculated from Reads Per Kilobase of transcript, per Million mapped reads (RPKM) of *argR* (top) and *rnc* (bottom) in methylation-active and passive hosts using RNA-seq utilised in this study (*hsdM* mutant). (D) Normalised gene expression values calculated from Reads Per Kilobase of transcript, per Million mapped reads (RPKM) of of *argR* (top) and *rnc* (bottom) gene expression in methylation-active and passive hosts (EcoKI mutant), analysis performed by Mehershahi and Chen (47).

Alt text: Graphs show the influence of EcoKI methylation on *argR* and *rnc* promoter activity across complementary experimental approaches. Data include *lux* reporter assays, qPCR measurements of *lux* transcription, and RNA-seq-derived expression values for *argR* and *rnc* in methylation-active and passive *E. coli* hosts, including datasets from the present study and a previously published EcoKI mutant study.

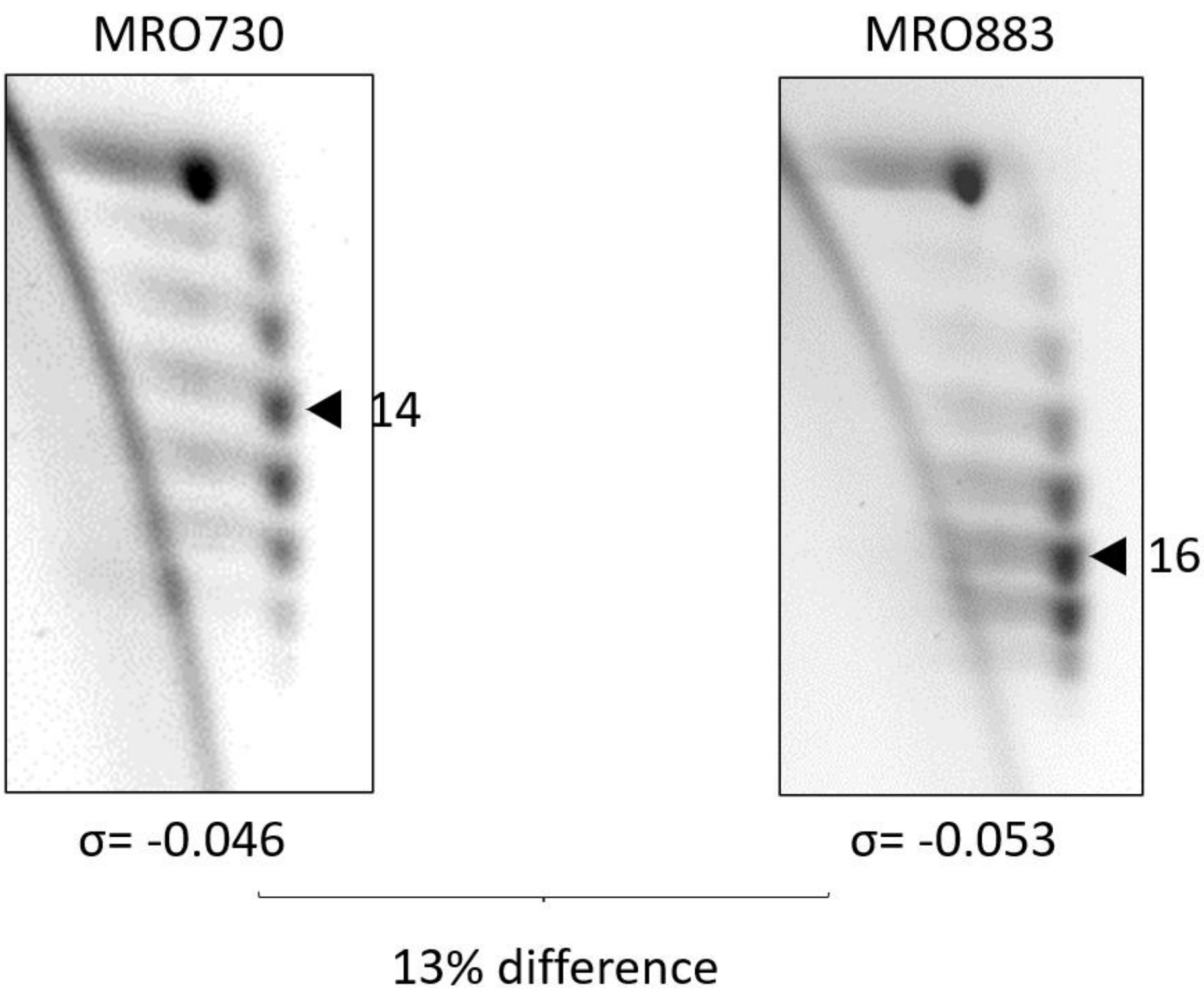

Figure S3: Two-dimensional chloroquine gels of native pDP1 plasmids isolated from MRO730 (SpnD39IIIE) and MRO883 (SpnD39IIIB) D39 strains. Black arrows indicate the most abundant topoisomers. Supercoiling densities were $\sigma = -0.046$ for MRO730 and $\sigma = -0.053$ for MRO883, corresponding to a 13% difference in supercoiling density. Supercoiling densities are shown as the σ value (Most abundant topoisomer/ Lk_0; Lk_0= size of the plasmid/ 10.5). Size of plasmid pDP1 is 3171 bp.

Alt text: Two-dimensional chloroquine gels showing methylation-altered plasmid supercoiling of native pDP1 plasmids isolated from MRO730 (SpnD39IIIE) and MRO883 (SpnD39IIIB) D39 strains
